# Animal welfare is a stronger determinant of public support for meat taxation than climate change mitigation in Germany

**DOI:** 10.1038/s43016-023-00696-y

**Published:** 2023-02-16

**Authors:** Grischa Perino, Henrike Schwickert

**Affiliations:** 1 Department of Socioeconomics, Universität Hamburg, Hamburg, Germany; 2 Center of Earth System Research and Sustainability (CEN), Universität Hamburg, Hamburg, Germany; 3 Center for Sustainable Society Research (CSS), Universität Hamburg, Hamburg, Germany

**Keywords:** Economics, Environmental studies, Psychology and behaviour, Climate-change policy, Social sciences

## Abstract

A tax on meat could help address the climate impact and animal welfare issues associated with the production of meat. Through a referendum choice experiment with more than 2,800 German citizens, we elicited support for a tax on meat by varying the following tax attributes: level and differentiation thereof, justification and salience of behavioural effects. Only at the lowest tax level tested do all tax variants receive support from most voters. Support is generally stronger if the tax is justified by animal welfare rather than climate change mitigation. Differentiated taxes that link the tax rate to the harmfulness of the product do not receive higher support than a uniform tax; this indifference is not driven by a failure to anticipate the differential impacts on consumption. While the introduction of meat taxation remains politically challenging, our results underscore the need for policymakers to clearly communicate underlying reasons for the tax and its intended behavioural effect.

## Abstract (web summary)

Meat taxes could help to induce dietary changes beneficial for the environment, animal welfare and public health. Through a referendum choice experiment, this study examines how level and differentiation of the tax, its justification and behavioural salience impact support for meat taxation in Germany.

## Main

The animal farming industry is in the public eye. Consumption and production of meat and dairy products and their consequences are discussed in society and politics alike^1–3^. The livestock sector accounts for 14.5% of all human-induced greenhouse gas (GHG) emissions^4^. Breeding and husbandry conditions, especially in intensive livestock farming, lead to animal diseases or painful disease-prevention measures such as tail-docking pigs^5,6^. Working conditions in meat processing firms have also drawn increasing attention of policymakers, which has partly resulted in legislative amendments^7–9^. From a health perspective, meat consumption levels are too high in industrialized nations, leading to increased risks for colorectal cancer and cardiovascular diseases^10^, and eventually straining public health systems^11,12^. Given the diverse deficiencies of the animal farming and meat production systems, policymakers are increasingly accounting for them, such as in the European Commission’s Farm to Fork strategy^13^. Alongside setting stricter rules and standards for producers, one potential intervention could be the introduction of a tax on meat and animal products. Modelling studies show that taxing meat and animal products could have strong steering effects, thus improving public health and reducing the environmental impact^14–20^.

In Germany, policymakers are discussing a tax on meat to address two of the issues named above, namely the climate and animal welfare aspects. In the context of introducing a carbon price for fossil fuels in the heating and transportation sector^21^, the German Green Party suggested a climate charge on animal products^22^. In addition, an expert commission set up by the then German Minister of Food and Agriculture suggested implementing a fixed animal welfare consumption tax, the so-called *Tierwohlabgabe*, on every kilogram of meat sold, with revenues intended to support farms in improving husbandry conditions^23^. In April 2022, the expert commission reminded the new government of its recommendation^24^. The climate change and animal welfare debates are conducted rather independently of one another, although they concern the same industry and the same products. We therefore focus on these two aspects while acknowledging that there are other reasons to motivate meat taxation such as biodiversity loss, water pollution and health concerns^11,25,26^.

The introduction of taxes on food is undoubtedly a political challenge, particularly in times of high inflation and globally rising food prices^27^. Numerous surveys and choice experiments have examined individuals’ preferences regarding (carbon) tax schemes in general, and animal products in particular^28–35^. Several policy characteristics have been found to increase public support, for example, refraining from calling the charge a tax, earmarking revenues, establishing progressive taxation and clearly explaining the tax’s impact^36,37^.

In this Article, we varied additional tax attributes to determine their impact on support for meat taxation. Motivated by the two justifications discussed by policymakers in Germany, we tested if support rates for a tax on meat differ depending on whether the tax is levied to mitigate climate change or to improve animal welfare. On the basis of previous findings on the effectiveness or stated importance of different reasons to reduce meat consumption^38–42^, we hypothesized that support is higher for a tax aiming to promote animal welfare.

In addition, we compared two versions of a per-unit excise tax varying in their degree of differentiation. The uniform variant charges a fixed amount on every kilogram of meat sold, independent from the meat’s carbon footprint or the husbandry conditions. Examples for such a tax type are the proposed *Tierwohlabgabe* of the German expert commission and the German electricity tax. The second variant is, in the spirit of a Pigouvian tax^43^, differentiated to represent differences in external damages associated with the product, such as alcohol or tobacco taxes and the German CO_2_ price on fuels. Meat types with a higher carbon footprint in case of a climate tax, or produced by farms with poorer husbandry conditions in case of an animal welfare tax, are charged a higher tax rate per kilogram than those with lower emissions or better husbandry conditions, respectively. The two tax types are expected to affect consumption differently. A uniform tax primarily reduces meat consumption overall as it does not change relative prices within meat categories^44^. A differentiated tax is expected to affect both the level as well as the composition of meat products consumed^45^. The latter is due to increased prices of products associated with higher damages to other human and non-human beings. The additional steering effect of a differentiated tax helps to reduce these damages and is hence typically considered to better improve human and animal welfare compared with a uniform tax. We tested whether voters appreciate the Pigouvian idea once all other tax attributes, including earmarking of revenues, are held constant.

We presumed voters’ perceptions of the tax’s impact on consumption patterns to affect support rates. While there are, a priori, no reasons to expect that the justification of a tax influences its impact on consumption patterns, we would anticipate such effects for the degree of differentiation. However, whether consumers anticipate this difference and how it might affect their stated support remains to be seen. Research on the acceptance of congestion charges, waste taxes and a carbon tax finds that trial periods increase support and people update their beliefs regarding the tax^36^. Thus, we tested whether varying the salience of expected behavioural effects on consumption affects support rates. If participants anticipate the stronger steering effect of a differentiated tax and appreciate it, then higher support rates would be expected if this is made more salient. We increased salience for a subgroup by asking participants to reflect upon the tax’s potential impact on consumption behaviour before eliciting their support.

We addressed all three attributes discussed above in a referendum choice experiment, in which a sample representative of the German adult online population was asked to vote on a tax on meat. The referendum setting was chosen as previous studies find that referendum surveys are externally valid^46–48^ and incentive compatible if perceived to be consequential^49,50^. To increase consequentiality, participants knew that referendum results of this study will be sent to the committees of the German parliament responsible for agriculture and the environment^51^, allowing policymakers to update their beliefs about public support for a tax on meat^52^. We randomly assigned participants to one of two tax purposes (animal welfare versus climate), one of two tax types (uniform versus differentiated tax) and one of two salience levels (low versus high salience of the tax’s effect), that is, eight treatment groups (Table 1 and Methods). Within subjects, proposals differed only in tax level, which was gradually increasing from the first to the last proposal. Participants had to make a decision on six consecutive proposals.

**Table 1:** Overview on exogenously varied attributes in the experiment

Characteristics	Variants	Implementation in experiment
Tax justification	Animal welfare	Animal welfare levy. Revenues used to improve animal welfare in livestock farming
	Climate	Climate levy. Revenues used to invest in climate protection
Degree of tax differentiation	Uniform	Equal amount per kilogram meat, independent from husbandry level or meat type (that is, climate impact)
	Differentiated	Differentiated amount, dependent on husbandry level or meat type—better rearing conditions/lower GHG emissions, lower levy
Salience of behavioural effects	Low	Question on expected behavioural response to proposed tax scheme after voting in the last referendum
	High	Question on expected behavioural response to proposed tax scheme before voting in the first referendum

Participants are randomly assigned to one of the two variants for each characteristic at the beginning of the experiment. In total, there are eight experimental groups. For further details, see Methods.

Our results contribute to the delicate topic of how to reduce meat consumption as one of the big societal, environmental and ethical challenges humanity faces^53^. As the paper focuses on public support and, in particular, on hypothetical voting in a referendum, the approach is, by design, anthropocentric, as only the preferences and values held by participants drive the results of the study. The paper is not concerned with why society should tax meat, but rather on how specific features, including justifications, affect support rates for such a tax. We add to the literature on instruments to influence meat consumption, more specifically on what affects people’s support for the rather heavy-handed fiscal intervention of a tax on meat. This complements studies looking into consumers’ preferences regarding information provision and labels on meat products^38,41,54^. By considering two different rationales for a tax on meat (climate protection versus animal welfare), we broaden research on the acceptance of carbon taxes^36^ by the animal welfare aspect. We thereby address different arguments for meat taxation as requested by Fesenfeld et al.^33^ and extend the findings by Fesenfeld et al.^25^ on willingness to pay for a tax for animal welfare, climate, local environment and health frames in Germany. Moreover, we complement the emerging literature on the link between tax support and use of tax revenues^55,56^. We also take current policy discussions into account by comparing a Pigouvian tax—which is usually favoured by economists^57^—to a uniform tax debated in Germany. Empirical evidence on whether support rates differ between a uniform and a differentiated tax on meat remains limited.

## Results

### Tax justification and level drive support rates

We tested pre-registered hypotheses on the impact of the attributes of a tax on meat on support by voters. The attributes considered are: tax level and differentiation thereof, justification and salience. As in a real referendum, we counted only valid responses, that is, Yes and No votes. The support rate thus equals the share of Yes votes among valid votes. Rates of abstention are similar across all tax levels and schemes, ranging from 6% to 8% (Supplementary Fig. 4).

**Fig. 1:**
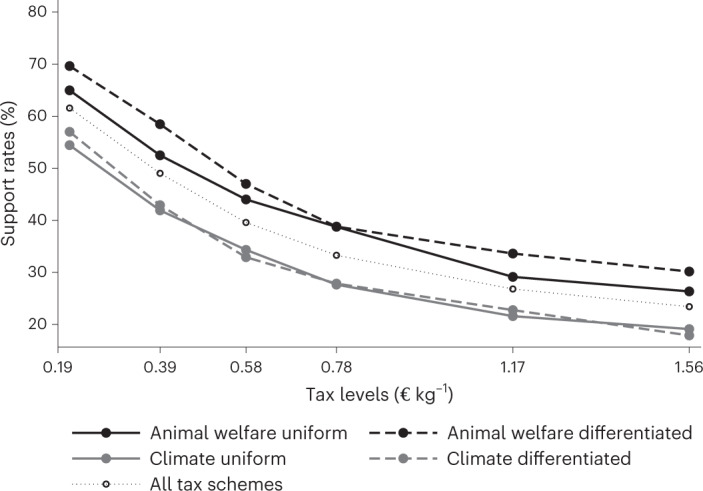
Support for of meat tax on meat across tax schemes. Data points show the percentage of participants who chose ‘Yes, I vote for the introduction of this levy.’ at each tax level proposed by tax scheme. Tax justifications are distinguished by line colour (animal welfare versus climate) and degree of differentiation by line style (uniform versus differentiated). High and low salience groups are pooled. The lowest tax level corresponds to a carbon price of €25 t^−1^ CO_2_, the highest tax level to €200 t^−1^ CO_2_. Tax levels in € kg^−1^ at a support rate of 50% of valid votes were as follows: animal welfare uniform, 0.4443; animal welfare differentiated, 0.5291; climate uniform, 0.2598; climate differentiated, 0.2885; and all tax schemes, 0.3734. Values are derived by linear interpolation using average support rates per tax scheme as depicted in the graph.

**Fig. 2:**
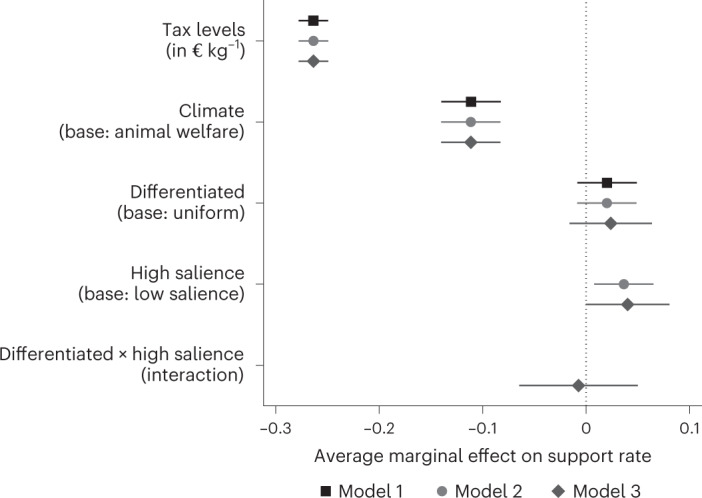
Effect of tax characteristics and salience on support for tax on meat tax. Data points indicate mean percentage point estimates with cluster-robust 95% CIs from linear regressions of valid votes for a proposed tax scheme (1 for yes, 0 for no) for *n* = 15,908 observations (corresponding to 2,759 respondents). Independent variables are tax levels in € kg^−1^ (continuous), tax justification (dummy variable: 0 for animal welfare, 1 for climate), degree of differentiation (dummy variable: 0 for uniform, 1 for differentiated tax) and salience (dummy variable: 0 for low salience or belief elicitation task after referendum task, 1 for high salience or belief elicitation task before referendum task). Model 1 comprises independent variables tax levels, tax justification and degree of differentiation. In model 2, salience is added. Model 3 comprises all independent variables mentioned, including the interaction term between degree of differentiation and salience. Robustness checks including control variables on demographics, political views, consumption habits, consequentiality perceptions and attention indicators do not change estimates (Supplementary Fig. 5).

Figure 1 shows support rates by tax scheme at each of the six average tax levels proposed, as well as projections at which tax level a particular variant would just pass the referendum. We conducted a set of linear regressions with the binary outcome variable 0 representing refusal of the proposal and 1 representing support. Average marginal effects of all attributes are shown in Fig. 2. For ease of interpretation, we set the tax-level variable to be continuous, which implies a linear relationship. We are aware that this is only an approximation and that the true relationship between tax levels and support rates might be non-linear^33,58^. As robustness checks, we ran all models with tax levels as categorical variables and logistic regressions due to the binary nature of the dependent variable. Coefficient estimates and statistical significance do not change considerably (Supplementary Fig. 6 and Supplementary Tables 5 and 6).

The percentage of votes in favour of the proposed tax on meat monotonically decreases by 2.6 percentage points (here, and in the following, we report 95% confidence intervals (CI) from model 3 in Fig. 2 (−2.49 pp, −2.78 pp)) for each €0.10 kg^−1^ increase in the tax rate. The average support rate is 62% at the lowest tax level of €0.19 kg^−1^, corresponding to a carbon price of €25 t^−1^ CO_2_. At this level only, every proposed tax scheme would receive a simple majority. Support monotonically decreases in the tax rate and reaches on average 23% at the highest tax rate of €1.56 kg^−1^, corresponding to €200 t^−1^ CO_2_. This confirms our hypothesis that support is decreasing in the tax level. Fifty per cent of participants would still support a tax level of €0.39 kg^−1^ if linearly interpolated (Fig. 1).

Support for climate-justified taxes is significantly lower than for otherwise identical animal welfare-justified taxes across all tax levels. On average, an animal welfare tax receives 11.1 percentage points (8.3 pp, 14.0 pp) more Yes votes than an otherwise identical carbon tax. This again is in line with the pre-registered hypothesis. All estimates are similar and highly statistically significant across models. Interestingly, the degree of differentiation of the tax has at most a minor and statistically not significant impact on support rates (*β* = 0.024, (−1.6 pp, 6.4 pp)), which counters our hypothesis.

High salience increases the support rate by 4.0 percentage points (0.0 pp, 8.1 pp). Participants who were induced to think about the potential effect of the proposed tax before they vote are thus more likely to support the scheme. However, we find no significant interaction between salience and the degree of differentiation (*β* = −0.007, (−6.5 pp, 5.0 pp)). The interaction term is close to zero and statistically insignificant. Counter to our pre-registered hypothesis, the effect of a differentiated tax is not more pronounced in the case of high salience.

### Expected tax impact varies by justification and differentiation

**Fig. 3:**
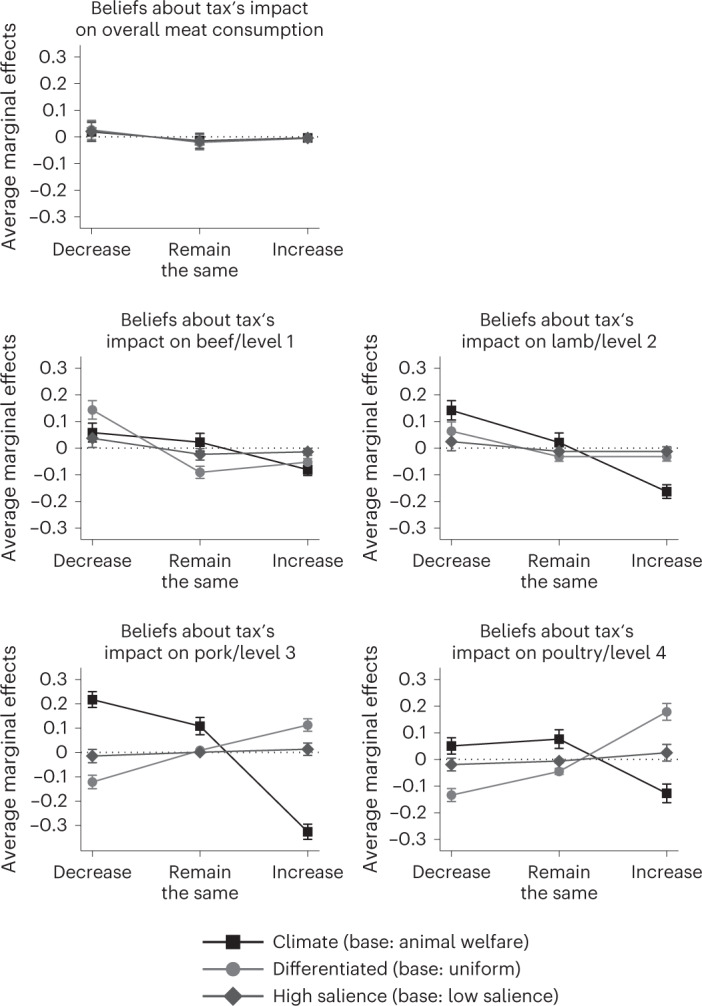
Effects of tax attributes and salience of beliefs re. development of meat consumption overall and per subcategory. Data points indicate average marginal effects with cluster-robust 95% CIs from generalized ordered logistic regression of beliefs regarding development of meat consumption with answer levels (1) decrease, (2) remain the same or (3) increase for *n* = 2,855 respondents. Independent variables are tax justification (dummy variable: 0 for animal welfare, 1 for climate), degree of differentiation (dummy variable: 0 for uniform, 1 for differentiated tax) and salience (dummy variable: 0 for low salience or belief elicitation task after referendum task, 1 for high salience or belief elicitation task before referendum task).

We conducted an analysis of participants’ beliefs about the behavioural impacts of the tax schemes. This analysis is exploratory given the hypotheses tested were not pre-registered. It aims at providing insights on what might drive the main results presented in the previous section. Participants stated their expectation about the market-wide development of meat consumption if the proposed tax scheme was to be implemented. Figure 3 shows average marginal effects on the probability of choosing the three possible answer categories (decrease, remain the same or increase) from generalized ordered logistic regressions for overall meat consumption and consumption in the subcategories beef/husbandry level 1, lamb/husbandry level 2, pork/husbandry level 3 and poultry/husbandry level 4, respectively.

Looking at tax types, we find that participants expect the differentiated tax to be significantly more effective in steering meat consumption towards lower-impact meat compared with the uniform one. For the two meat types/husbandry levels that are taxed the most under a differentiated tax, the probability of choosing ‘decrease’ is significantly higher for those facing a differentiated rather than a uniform tax. The opposite applies for the two meat types/husbandry levels that are taxed the least under a differentiated tax. Looking at answer option ‘increase’, the marginal effects are reversed. In addition, participants expect overall meat consumption not to be impacted by the degree of differentiation, which is consistent if effects from the four subcategories cancel each other out.

**Extended Data Fig. 1:**
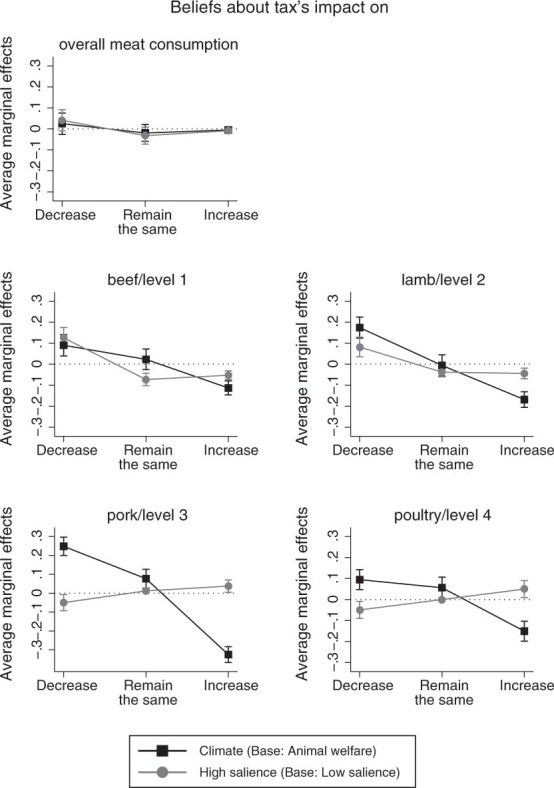
Effects of tax characteristics and salience of beliefs re. development of meat consumption overall and per sub-category - Uniform tax only. Datapoints indicate average marginal effects with cluster-robust 95% confidence intervals from generalized ordered logistic regression of beliefs regarding development of meat consumption with answer levels (1) decrease, (2) remain the same, (3) increase for *n* = 1,430 respondents. Only participants who saw a uniform meat tax are considered. Independent variables are tax justification (dummy variable: 0 for animal welfare, 1 for climate) and salience (dummy variable: 0 for low salience, 1 for high salience).

Regarding the tax’s justification, we find that participants expect the climate tax to be significantly more likely to decrease consumption in all meat type/husbandry level subcategories compared with the animal welfare tax. Even if we look at the uniform tax subsample only, we find the same differences (Extended Data Fig. 1). For a uniform tax, prices of all meat products on the market rise by the same amount, independent of whether the levy is raised for climate or animal welfare purposes. Thus, effects cannot be driven by perceived or real differences in the market shares of husbandry/meat type categories or different degrees of substitutability between them. Moreover, participants do not expect a significantly different effect of the climate tax compared with the animal welfare tax for overall meat consumption, which contradicts responses for the subcategories of consumption.

## Discussion

Our study provides important insights for policymakers on how to design a tax on meat to receive public support. First, supported tax levels are found to be rather low in our experiment. At the lowest tested tax rate of, on average, €0.19 kg^−1^ (equivalent to €25 t^−1^ CO_2_), a simple majority of participants votes in favour of a tax on meat in every tax scheme suggested. For the second lowest tax rate of, on average, €0.39 kg^−1^ (or €50 t^−1^ CO_2_), only taxes justified by animal welfare win a referendum. This level of an animal welfare tax matches the proposal by the expert commission reporting to the previous German government^23^. Thus, the proposal is backed by voters at the time of the experiment. We acknowledge that support for the actual tax rates tested represents a snapshot given participants’ current disposable income, recent societal debates and other structural and individual factors. Nonetheless, given that the rate of support for a tax on meat is strongly decreasing, in particular, at the lower end of the range tested in our study as well as in the extant literature^33,58^, we recommend starting with a low rate when introducing a tax on meat. Following a ratcheting-up strategy^59^ is likely to receive more support than trying to go full scale initially. However, more research is needed to determine the exact relationship between (dynamic) tax rates and public support.

We find that participants are more willing to vote for a tax if its purpose is to improve animal welfare as opposed to reducing the climate impact of meat products. This complements results from (choice) experiments and surveys on labels and information provision, in which animal welfare arguments are found to be more important or effective in inducing intrinsically motivated behavioural change than climate protection arguments^38–42,60–63^. The stronger appeal of animal welfare motives is also present in the context of the more intrusive intervention of a tax on meat. Our result is, however, in contrast to Fesenfeld et al.^25^ who find no significant differences between the two framings. This difference in findings might be driven by the naming and description of the tax schemes in the two studies. They tested how different independent frames (climate change mitigation, animal welfare and health benefits) affect support for a tax on meat. In contrast, we made the frame explicit in the tax name, calling it ‘animal welfare’ or ‘climate levy’. The explicit framing in the tax name might send a more credible signal to participants that animal welfare is actually addressed with the tax, increasing support. Moreover, the lack of information in Fesenfeld et al. on how the tax revenues would be spent might have substantially reduced support for a tax in their study, and hence made it more difficult to detect differences between frames. In contrast, we stated that tax revenues are earmarked and provided detailed information on which meat types or animal welfare levels are taxed and why. Especially for animal welfare, voters might be more supportive if they have a concrete idea of how animals might benefit from a tax. While earmarking seems to be less important when considering combined support of several food policies^33^, it is found to be a crucial success factor for acceptance of a stand-alone carbon tax^36,64,65^, and hence maybe also for a stand-alone tax on meat. The design of our study does not allow distinguishing between the framing and the earmarking aspect. Specifying their relative importance is left for future research.

Surprisingly, participants seem to attribute a stronger steering effect to a climate tax compared with an animal welfare tax, even if they are identical in all other respects. We can only hypothesize why this is the case. Preferences for animal welfare taxes might not be driven by beliefs in their ability to reduce meat consumption, but potentially by beliefs in their effectiveness of promoting animal welfare independent of the amount of meat consumed. This is plausible if consumers consider the lives of farm animals to be worth living and are not primarily concerned about the fact that animals have to be killed to produce meat^66^. Moreover, participants might expect additional individual benefits from paying an animal welfare tax because they associate healthier or tastier products with higher animal welfare standards. In the latter case, participants would consider animal welfare not only as a public good^67^, but would also derive private benefits from improving rearing conditions (for similar thoughts regarding labelling antibiotic use on meat products, see refs. ^41,60^). Future research could look into drivers behind preferences for an animal welfare tax. For policymakers, this shows that justifications matter, potentially more so than expected impacts on behaviour. Our study does not shed light on the question of whether combining justifications (and splitting revenues) would improve or weaken support for the measure.

Our findings show that the degree of differentiation does not play an important role in shaping support for a tax on meat. Simulation studies in other contexts, namely sugar-sweetened beverages, suggest that a differentiated tax is more effective in reducing externalities^68–71^. As answers to the belief questions show, participants on average understand the mechanism behind a differentiated tax and also expect a stronger steering effect from this tax type. However, we only find a minor and mostly statistically insignificant positive effect on support compared with a uniform tax. Raising the salience of the stronger steering effect has no impact on support rates. We conclude that voters might well understand that Pigouvian taxes are more effective in changing consumption patterns than uniform ones, but that they do not appreciate this. This finding is in line with empirical results by Kallbekken et al.^72^ who find that support rates for a Pigouvian tax in a laboratory experiment do not increase if participants are informed about its benefits. Our results confirm their findings and extend them in two directions. The lack of a significant interaction effect between raising the salience of a proposed tax scheme and the degree of differentiation is analogous to their observation that educating participants about the additional steering effect does not systematically change support rates. This builds our first extension, that is, that, on average, participants are able to qualitatively anticipate the steering effect of differentiated taxes in a more complex real-world setting without being educated about them by the experimenter. Second, we directly compare support for a Pigouvian with support for a uniform tax. Our results show that adding a steering effect does not increase support rates compared with a tax that is identical in all other features. Overall, the results substantiate the point that the indifference found between uniform and differentiated taxes is not primarily driven by participants who do not understand how the tax schemes differ, but it is rather caused by a lack of caring about this difference. This provides relevant insights for policymakers. The indifference between Pigouvian and uniform tax is at least partially good news for them as there is low risk in implementing the more effective differentiated tax. The recommendation is weakly backed by comments in the Remarks fields of our survey. Thirty-eight participants who had been assigned to a uniform tax treatment criticize the lack of differentiation or state that they would prefer a differentiated tax. On the other hand, only one participant in the differentiated treatments asks for a uniform tax.

Given that we find a positive effect of high salience on support rates, we additionally recommend communicating the tax’s desired behavioural impact very clearly to win the public over. Our result supports previous findings that experiencing the effect of a tax in trial periods makes people more likely to support it^36^ if thinking the effect through is indeed a proxy for such a trial experience.

To conclude, there is support for a tax on meat in Germany, but only under certain conditions that policymakers would benefit from taking into account. The version recently suggested by a government-installed expert commission meets these criteria, but more effective taxes would also be supported by voters. While we focus on Germany, other countries have been, or are currently, discussing different forms of a tax on meat as well. In October 2022, New Zealand’s government proposed to price livestock emissions at the farm level with revenues used to support farmers in their efforts to reduce emissions^73^. This corresponds to the differentiated tax treatment in our study as the price impact will differ in line with the emission intensity of meat types. In the Netherlands, policymakers presented concrete proposals to implement a tax but so far have not been able to convince a majority in parliament^74^. In the UK, meat taxation was discussed, but despite being found to have a substantial potential impact on GHG emissions and public health^26^, it was explicitly disregarded from the National Food Strategy published in 2021 due to potential lack of acceptance among citizens^75^. The Danish Council of Ethics, a Danish think tank, recommended a tax on red meat for Denmark in 2016, which was refused by politicians^76,77^. Our findings could be particularly relevant for the failed proposals by checking if the taxes could have been defined or framed differently. Future research could leverage our design and compare support rates internationally.

## Methods

We developed a referendum choice experiment to elicit support for a tax on meat. We ran it through an online survey with 2,855 participants. The survey was pre-registered on the American Economic Association’s registry for Randomized Controlled Trials registry with ID AEARCTR-0008507 and conducted between 30 November and 9 December 2021. The sample was recruited by a professional panel provider (respondi AG). All participants were informed about and consented to their answers being used for scientific purposes only.

### Experimental design

Supplementary Fig. 1 provides an overview on the online survey and experimental design. The survey was programmed by us with Lighthouse Studio 9.11.0 by Sawtooth Software and hosted on Sawtooth Software server. After collecting demographics, information on political positions and food consumption behaviour, we informed survey participants that we would like to know their opinion on the introduction of a charge on meat products in Germany. Following this general statement, we randomly assigned participants to one of two tax justifications, one of two degrees of differentiation and one of the two salience levels (as presented in Table 1). Respondents then received a detailed explanation of a proposal for the respective tax they were assigned. In each group, the proposal stated that the government would introduce a levy on meat products, namely fresh meat, sausages and cold cuts, and that the charge would be levied on each kilogram of meat sold, increasing prices for consumers. It subsequently contained detailed information depending on the assigned tax scheme:

i. **Name of the levy:** animal welfare levy or climate levy. The word levy (*Abgabe* in German) was explicitly chosen to avoid negative connotations with the word tax (*Steuer* in German), but also to refer to the term already used in the public discourse for the animal welfare levy.
ii. **Justification of the levy:** related to either the husbandry system or the GHG emissions of meat. For both reasons, we detailed which husbandry systems/meat types are considered. For the animal welfare levy, it would be the so-called *Haltungsformstufen* (levels of husbandry system) from levels 1 to 4, with 4 being the level with the best rearing conditions. We are thereby specifically adopting an existing German label for husbandry and animal welfare conditions that has been developed by major German supermarket chains. More information on the *Haltungsform* label can be found at www.haltungsform.de. This voluntary label is not applied to all meat products in the market, but only to an arbitrary selection in participating supermarkets. For the climate levy, the main meat types consumed in Germany were considered: beef, lamb, pork and poultry.
iii. **Type of the levy:** uniform/equal or differentiated/dependent on husbandry system/meat type. We explained that the levy would be either the same for all husbandry systems or meat types or depend on the latter in that the better the rearing conditions or the lower GHG emissions, the lower the charge. We refrained from mentioning actual tax rates here to avoid any anchoring effect, especially with regard to the belief elicitation task. However, to make the tax type clear to participants, we added a graph depicting the ratio between tax levels for the four systems or meat types, respectively (for examples, see Supplementary Fig. 2). Although the illustrative graphs do not contain actual numbers, the ratios of the bars to each other match actual tax levels used in the referendum. The derivation of tax levels is explained in ‘Calculation of tax levels’.
iv. **Revenues from the levy:** investment in improvement of animal welfare in livestock farming or in climate protection. We explicitly mentioned that revenues from the tax would be earmarked to the respective tax justification since previous literature detects earmarking as important for tax support^36^.

In addition, participants could choose to open a detailed document containing information on the underlying criteria for the husbandry system levels or GHG emissions per meat type. It was measured if they requested the additional information and for how long they stayed on this information page. For the animal welfare tax schemes, the detailed criteria of the *Haltungsform* label were adopted and shown to participants^78^. For the climate levy, we reprocessed information from the 2.0 version of the Global Livestock Environmental Assessment Model of the Food and Agriculture Organization of the United Nations^79^. GHG emissions per meat type along the value chain of meat production were shown in a graph and also explained in detail.

After presenting the proposal, we explained the referendum set up to participants. They should vote on six different proposals that only differ in tax rate. We explicitly stated that participants should vote as if the proposal shown was the only one on the ballot. To increase consequentiality further, we told participants that we will send a letter with the summarized voting results to the committees of the German parliament responsible for agriculture and the environment^51^. An exemplary letter was added. We sent the letter with a description of the study, results and a link to the publicly available working paper to the two committees on 19 May 2022.

**Extended Data Fig. 2:**
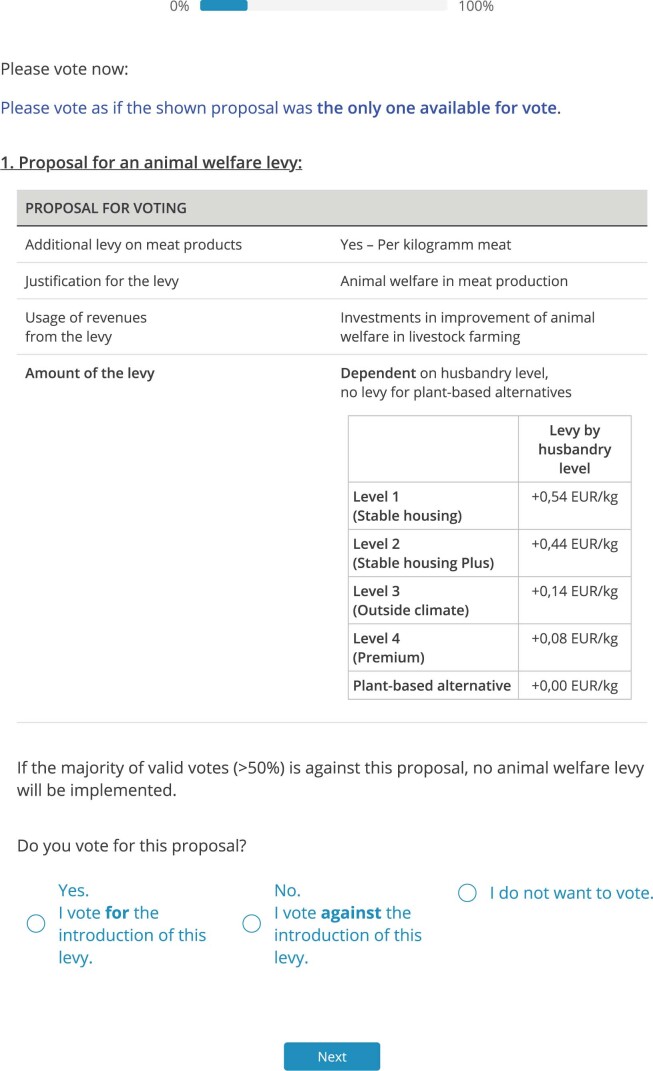
Example of choice set. Participants saw six of these choice sets with only tax levels increasing from lowest to highest. The tax characteristics remained the same for each participant.

In the following referendum task, participants were given the six choice sets. Each proposal contained all the information mentioned in the detailed explanation given upfront, but in an abbreviated form. In addition, an explicit tax rate was now given. An example of one choice task can be found in Extended Data Fig. 2. For each choice set, participants could pick one of three options:

1. Yes. I vote for the introduction of this levy.
2. No. I vote against the introduction of this levy.
3. I do not want to vote.

We explicitly listed the option to abstain to signal that, as in reality, participants are not forced to vote.

Moreover, participants had to perform a belief elicitation task. Depending on their random assignment, they received the task after (low-salience group) or before the referendum task (high-salience group). Respondents were first asked how they would expect the overall meat consumption to change if the respective tax scheme was introduced. They could choose between three options: overall meat consumption will (1) decrease, (2) remain the same or (3) increase. Then, participants were asked four subquestions on their expectations regarding the change in consumption by husbandry level (levels 1–4) or meat type (beef, lamb, pork and poultry). In addition, we requested respondents’ beliefs pertaining to other participants’ answers on the previous five questions.

The survey concluded with items to control for social desirability bias^80^ and questions on the perception of consequentiality^51^, that is, if respondents think the government will and should take their votes on this survey into account.

### Calculation of tax levels

A crucial element of the experiment is the meat tax levels participants must vote on. Apart from learning more about the importance of the tax scheme’s characteristics for voters, we also want to find out which price premium they would accept. The proposed rates increase from the first to the last choice set, starting at on average €0.19 kg^−1^ of meat and gradually rising to on average €1.56 kg^−1^. These levels are not arbitrarily chosen, but based on GHG emissions of the different meat types and varying CO_2_ prices. For the lowest carbon price, we chose €25 t^−1^ CO_2_, equal to the German carbon price introduced on fossil fuels not covered by the European Union Emissions Trading System in 2021. For the highest price, we picked €200 t^−1^ CO_2_ because the German Environment Agency (*Umweltbundesamt*) estimates the social costs of carbon per ton of CO_2_ to be at this level^81^. Calculations to derive the six tax levels are depicted in Supplementary Fig. 3.

We chose to base the tax levels on underlying GHG emissions because quantifying the marginal damages associated with GHG emissions is well established, albeit controversial. A real carbon tax for meat would be based on similar logic, taking emissions of the meat production process into account. From these calculations, we directly confirmed the tax levels for our Climate Differentiated tax scheme. For the Climate Uniform scheme, we calculated a weighted average by multiplying the tax rate per meat type by the type’s 2020 share of total meat consumption. Repeating this procedure for each carbon price level generated the level of uniform tax for each of the CO_2_ prices respectively.

For animal welfare, there is not yet a comparable and established procedure to monetize marginal damages for animal welfare. To allow for comparisons across treatment groups, we used the tax levels from the climate treatments and applied them to the animal welfare schemes. In case of the Animal welfare Differentiated tax scheme, we applied the meat type-specific tax levels to the four husbandry levels. The tax for the lowest husbandry level 1 equals the tax for beef, the second lowest husbandry level 2 is equal to the tax for lamb and so on. For the Animal welfare Uniform scheme, we used the same weighted average as for the Climate Uniform one. That way all tax rates presented to participants are identical across schemes. Differences in support rates can hence be attributed to the justification provided.

### Sample

Our survey sample of 2,855 participants is drawn from the German adult population. The survey was fully completed by 3,169 participants. We excluded 314 respondents whose survey time was below the 5th (less than 5 min) and above the 95th percentile (more than 45 min) to account for inattention. Supplementary Table 7 shows that our main results are robust against this restriction. Effect sizes are marginally lower, but qualitatively the same. Median survey time is 12.4 min. Respondents were compensated for their time at the standard rate of the professional panel provider. They could also receive an additional bonus payment for the belief task if their guess of what other survey participants answered was sufficiently close to the real value. Bonus payments were calculated on the basis of answers of the unrestricted sample of 3,169 respondents who fully completed the survey. Average bonus payment was €0.145. A bonus of at least €0.10 was received by 1,581 participants.

Supplementary Table 1 summarizes demographics of the restricted sample in column 1. Column 2 lists mean values for the German adult population. As *P* values in column 3 indicate, the sample is representative in terms of sex and region of residence on a federal state level. In terms of age, the youngest age group is minimally underrepresented in favour of the oldest respondents. The unrestricted sample, in which all complete surveys were considered, is also representative in terms of age. Moreover, monthly net household income is similar, but the sample is better educated compared with the overall population. Supplementary Table 2 provides more details on demographics in each of the eight experimental groups. We did not conduct treatment group balance checks because we consider our experiment to be a ‘clean’ one according to Mutz et al.^82^. They define a clean experiment as one in which the randomization mechanism used is not faulty and no differential attrition occurs. Only if either of these two conditions was not fulfilled do they recommend balance tests as a tool when analysing the data. We consider our randomization mechanism not to be faulty as a random number between one and eight (for in total eight experimental groups) has been generated by the survey software used. In addition, we checked for attrition between the pre- and the post-treatment sample and between the pre-treatment sample and each treatment group by comparing demographics age, sex, region of residence, net income and education. We do not find any significant differences on the 10% significance level. Hence, we refrained from conducting treatment group balance checks.

Although a tax on meat would affect only meat eaters, or rather meat buyers, we refrained from screening out vegetarians or vegans since they could all vote in a referendum. In fact, those groups might be the ones who care the most about animal welfare standards^83^. Seven per cent of all participants identified themselves as vegetarians or vegans, another 2% as pescatarians. These numbers are slightly below results from other German surveys (for example, 12% vegetarians and vegans^84^ or 10% vegetarians, vegans and pescatarians^85^). Since participants could also buy meat for their household and not consume it themselves, we asked for their meat purchasing behaviour as well. Only 5% of participants said that they never buy any of the meat types. Thus, almost our entire sample would be financially affected if a tax on meat was introduced.

### Statistical analysis

For all statistical analysis, we used the statistical software STATA (version 16.1). We have a total of 17,130 observed choices resulting from 2,855 participants each voting six times. For calculation of support rates, we considered only valid votes as in a real referendum. This restriction reduces observations to 15,908. We estimated ordinary least squares linear regressions to determine the effect of each tax characteristic and salience on support rates for the tax on meat. The outcome variable is support for the tax on meat, and it is binary, with 0 standing for refusal and 1 for support of the respective proposal. The tax-level variable was set to be continuous. The independent variables for the experimental groups are all binary: tax justification (0 for animal welfare, 1 for climate), degree of differentiation (0 for uniform, 1 for differentiated tax), salience (0 for low salience or belief elicitation task after referendum task, 1 for high salience or belief elicitation task before referendum task) and the interaction term between degree of differentiation and salience (1 for differentiated tax times high salience, 0 otherwise). Since each participant had to make six consecutive choices, we clustered standard errors by respondent. The results are shown in Fig. 2 and Supplementary Table 4. As robustness checks, we included control variables (Supplementary Fig. 5 and Supplementary Table 4). Control variables are demographics as shown in Supplementary Table 2 as well as views on government (perception of governmental involvement on a seven-point Likert scale from 1 for ‘government is doing too much’ to 7 for ‘government is doing too little’; trust in government on a seven-point Likert scale from 1 for ‘very low’ to 7 for ‘very high’), political positions (0 for left, 1 for middle, 2 for right, 3 for n/a, i.e. not specified by the participant), voting for Green party (0 for no, 1 for yes), identifying as pescatarian or vegetarian or vegan (0 for no, 1 for yes), consuming meat (0 for ‘eats meat’, 1 for ‘eats no meat’), buying meat (0 for ‘buys meat’, 1 for ‘buys no meat’), purchase frequencies by meat types beef, pork, poultry, lamb and others (six-point scale from 1 for ‘several times per week’ to 6 for ‘never’), frequency of buying organic meat (0 for ‘buys sometimes organic or less often’, 1 ‘buys organic rather often to always’, 2 n/a), importance of animal welfare or climate or organic among purchases (seven-point Likert scale from 1 for ‘not important at all’ to 7 for ‘very important’), consequentiality perception (politicians will consider survey results and politicians should consider survey results both on seven-point Likert scale from 1 ‘not agree at all’ to 7 ‘fully agree’), social desirability bias^80^ (six items grouped into self-deceptive enhancement and impression management—recoded to dummy variables for highest manifestation: 0 for no, 1 for yes, that is, social desirability bias) and attention (0 for ‘no correct answers to two attention questions’, 1 for ‘one of two’ and 2 for ‘two of two correct answers’). We also ran the same ordinary least squares linear regression models with the tax levels as six binary variables, omitting the lowest level as the base category (Supplementary Fig. 6 and Supplementary Table 5) and logistic regression models due to the binary nature of the dependent variable (Supplementary Table 6).

In the exploratory analysis, we analysed participants’ answers in the belief elicitation tasks. We estimated generalized ordered logistic regressions of beliefs regarding the development of overall meat consumption and the development of consumption in each subcategory (beef/level 1, lamb/level 2, pork/level 3 and poultry/level 4) on the tax characteristics and salience. The outcome variable of beliefs has three levels (decrease, remain the same and increase). We estimated generalized ordered logistic regressions with robust standard errors and then calculated estimated average marginal effects. They indicate, for each answer level, by how much the probability of choosing this answer level changes given the level of the respective independent variable. The number of observations in these models is equal to the number of respondents, that is, 2,855, since each participant performed the belief elicitation task once. Results are shown in Fig. 3 and Supplementary Table 8. We also ran a robustness check reducing the sample to participants who received the proposal of a uniform tax only, reducing observations to 1,430. Results are shown in Extended Data Fig. 1 and Supplementary Table 10.

### Reporting summary

Further information on research design is available in the Nature Portfolio Reporting Summary linked to this article.

## Supplementary information

Supplementary Information Supplementary Figs. 1–6, Tables 1–11 and survey questionnaire translated into English.

Reporting Summary

## Data Availability

Data and survey questionnaires are publicly available at Harvard Dataverse: 10.7910/DVN/YNMG1R. For calculation of tax levels (Supplementary Fig. 3), publicly available datasets were used. As source for emission intensities, we used the Global Livestock Environmental Assessment Model (version 2.0) by FAO^79^. As source for meat consumption by meat type, we used the 2021 report on market and supply situation with meat by BLE^86^. The original data and the corresponding tax level calculations are available in the Excel file ‘Calculation of tax levels’ at Harvard Dataverse.

## Code availability

The statistical analysis codes for replicating the results presented in the figures, tables and Supplementary Information are publicly available at Harvard Dataverse: 10.7910/DVN/YNMG1R.
