# Supplementary material for: Animal welfare is a stronger determinant of public support for meat taxation than climate change mitigation in Germany
Source: Nat Food. 2023 Feb 16;4(2):160–9. doi: 10.1038/s43016-023-00696-y (PMC10154199; doi:10.1038/s43016-023-00696-y)
Supplement: Supplementary file 1 — Supplementary Figs. 1–6, Tables 1–11 and survey questionnaire translated into English. [file 43016_2023_696_MOESM1_ESM.pdf]

nature food

Article

https://doi.org/10.1038/s43016-023-00696-y

Animal welfare is a stronger determinant of public support for meat taxation than climate change mitigation in Germany

In the format provided by the authors and unedited

Supplementary Information for
"Animal welfare is a stronger determinant of public support for meat taxation than climate change mitigation in Germany"

Grischa Perino* Henrike Schwickert†‡

Contents

1 Additional material on experimental design 2

2 Sample statistics 5

3 Robustness checks 7

4 Regression tables 10

5 Survey questionnaire 18

*Department of Socioeconomics and Center of Earth System Research and Sustainability (CEN), Universität Hamburg, Germany.

†Department of Socioeconomics, Universität Hamburg.

‡Corresponding author: henrike.schwickert@studium.uni-hamburg.de

1 Additional material on experimental design

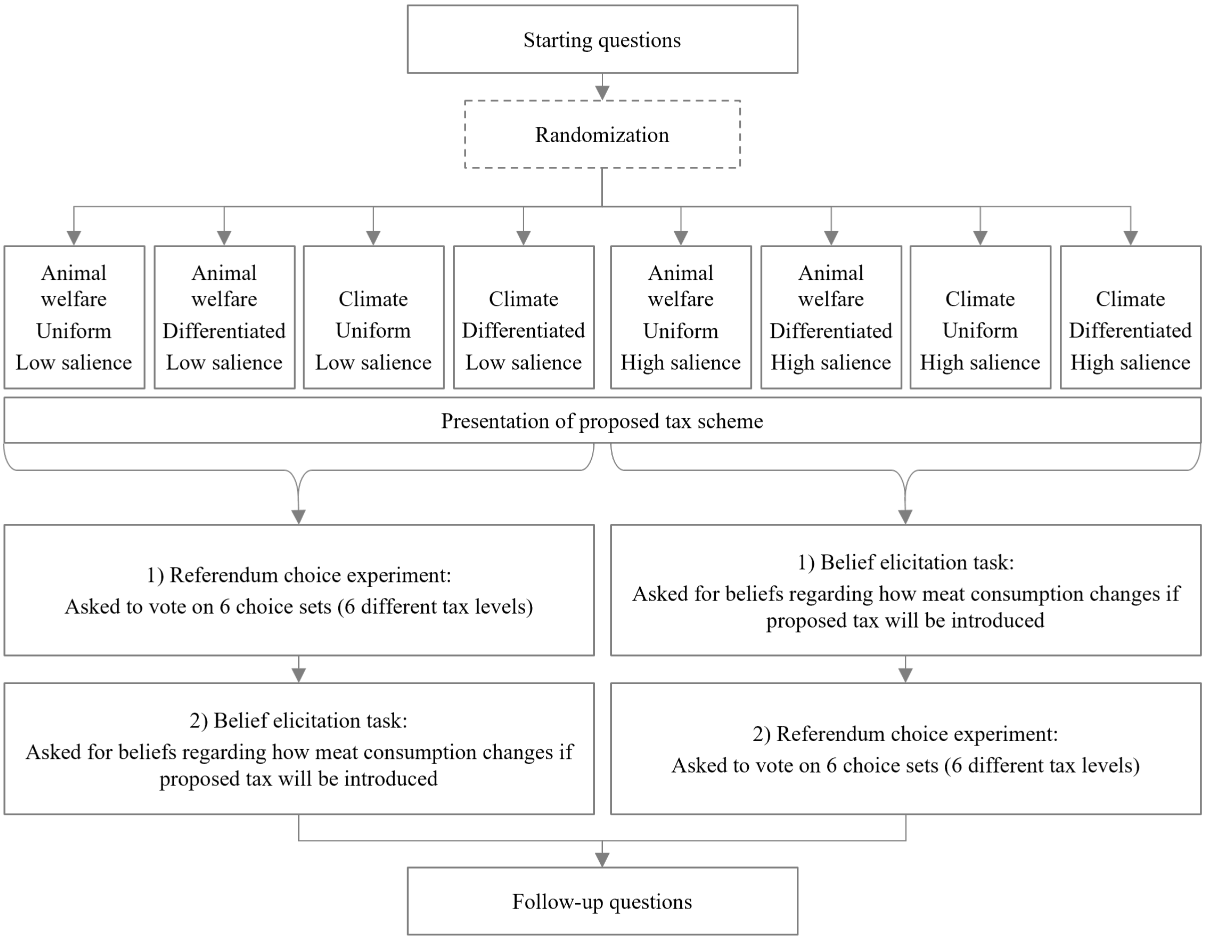

Supplementary Figure 1: Overview on experimental design. Schematic overview on survey flow and eight experimental groups.

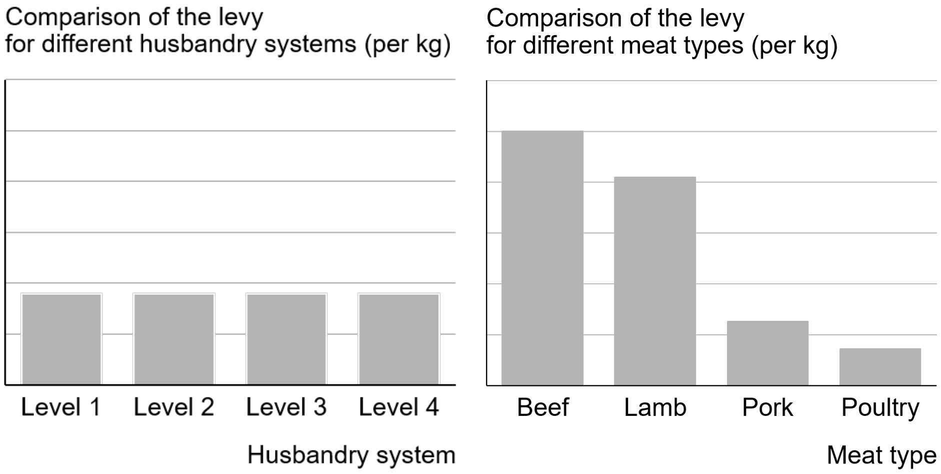

(a) In *Animal welfare Uniform* tax scheme

(b) In *Climate Differentiated* tax scheme

Supplementary Figure 2: Examples of illustrative graphs in the tax scheme description.

1. Differentiated tax rate:

Emission intensities *in t CO_2e/kg meat*	×	6 carbon prices *in €/t CO_2e*	=	Tax rate by meat type *in €/kg meat*

- GHG emissions along the value chain of meat production
- Emission categories considered:
  - Feed (CH_4)
  - Feed: fertilizer and crop residues (N_2O)
  - Feed: applied and deposited manure (N_2O)
  - Land use change (LUC): soybean and palm (CO_2)
  - LUC: pasture expansion (CO_2)
  - Enteric fermentation (CH_4)
  - Manure management (CH_4)
  - Manure management (N_2O)
- Region considered:
  - Western Europe
- Animal species considered:
  - Cattle
  - Sheep/Goats
  - Pigs
  - Chicken
- Commodity considered:
  - Meat

List of carbon prices considered:

- €25
- €50
- €75
- €100
- €150
- €200

E.g. at carbon price of €25/t CO_2e:

- Beef: €0.54/kg
- Lamb: €0.44/kg
- Pork: €0.14/kg
- Poultry: €0.08/kg

Source: GLEAM

---

2. Uniform tax rate:

Meat consumption by meat type *in %*	×	Tax rate by meat type *in €/kg meat*	=	Uniform tax rate *in €/kg meat*

Values from 2020:

- Beef: 17%
- Lamb/Sheep/Goat: 1%
- Pork: 58%
- Poultry: 23%

E.g. at carbon price of €25/t CO_2e:

- Beef: €0.54/kg
- Lamb: €0.44/kg
- Pork: €0.14/kg
- Poultry: €0.08/kg

- Weighted average: €0.19/kg

Source: Versorgungsbilanz 2020

Supplementary Figure 3: Calculation of tax levels. Source for emission intensities: GLEAM 2017 (FAO 2017. Global Livestock Environmental Assessment Model. Version 2.0.); source for meat consumption by meat type: Versorgungsbilanz 2020 (BLE 2021. Fleisch und Geflügel - Versorgungsbilanz). The original data and the corresponding tax level calculations are available in the Excel file "Calculation of tax levels".

2 Sample statistics

	Sample	German population	p-values
Female	50.4	50.2	0.799
**Age groups**			
Age <40 years	34.0	36.9	0.001
Age 40-59 years	39.5	39.4	0.935
Age 60+ years	26.5	23.7	0.001
**Region**			
North	18.2	18.0	0.731
East	17.0	17.3	0.694
South	29.0	29.3	0.695
West	35.8	35.3	0.607
**Education**			
Low	32.6	32.0	0.513
Middle	26.3	32.0	0.000
High	41.1	35.0	0.000
**Net household income**			
<€2000	28.4	27.0	0.096
€2000-3999	43.3	45.0	0.066
€4000+	28.3	28.0	0.721

Supplementary Table 1: Sample characteristics and comparison with German adult population. Column 1 shows means for the restricted survey sample of 2855 respondents, column 2 statistics for the German adult population. Data on sex, age and region of living on federal state level are from the Federal Statistical Office of Germany for December 2020. Data on education and income are from the best for planning 2020 IIII database. Federal states are pooled to regions. Education refers to highest educational qualification: Low = not graduated (yet), certificate of general secondary education; Middle = certificate of intermediate secondary education; High = university-entrance diploma, university degree/PhD. Net household income refers to monthly values. p-values in column 3 refer to paired two-sided t-tests.

	Animal welfare Uniform Low	Animal welfare Uniform High	Animal welfare Diff Low	Animal welfare Diff High	Climate Uniform Low	Climate Uniform High	Climate Diff Low	Climate Diff High	Total
Female	0.50	0.47	0.51	0.49	0.54	0.50	0.52	0.51	0.50
**Age groups**									
<40 years	0.36	0.37	0.34	0.34	0.33	0.32	0.34	0.30	0.34
40-59 years	0.39	0.36	0.41	0.37	0.40	0.40	0.38	0.44	0.40
60+ years	0.24	0.27	0.25	0.28	0.26	0.27	0.28	0.26	0.27
**Region**									
North	0.20	0.20	0.16	0.19	0.16	0.18	0.18	0.19	0.18
East	0.18	0.16	0.18	0.14	0.18	0.17	0.19	0.16	0.17
South	0.27	0.26	0.30	0.34	0.29	0.30	0.28	0.28	0.29
West	0.36	0.38	0.36	0.33	0.38	0.34	0.35	0.36	0.36
**Education**									
Low	0.33	0.36	0.33	0.32	0.28	0.34	0.33	0.31	0.33
Middle	0.27	0.26	0.27	0.27	0.28	0.25	0.24	0.26	0.26
High	0.40	0.38	0.40	0.41	0.44	0.41	0.42	0.43	0.41
**Net household income**									
<€2000	0.29	0.29	0.28	0.29	0.25	0.29	0.26	0.30	0.28
€2000-3999	0.45	0.44	0.45	0.41	0.44	0.38	0.46	0.43	0.43
€4000+	0.26	0.28	0.27	0.29	0.31	0.32	0.28	0.27	0.28
No. of adults in household	2.00	2.06	1.94	2.06	2.11	2.03	2.04	1.90	2.02
No. of kids in household	0.40	0.35	0.35	0.37	0.38	0.40	0.39	0.42	0.38
Have kids	0.56	0.55	0.55	0.59	0.55	0.60	0.56	0.57	0.56
Observations	354	360	361	355	359	350	342	359	2,840

Supplementary Table 2: Demographics by experimental group. The table shows the means of each dummy variable by experimental group and for the entire sample in the last column. Observations for the full sample are lower because 15 participants chose not to report if they have kids.

Tax scheme	Justification	Degree of differentiation	Low salience	High salience	Total
1	Animal welfare	Uniform	355	362	717
2	Animal welfare	Differentiated	364	358	722
3	Climate	Uniform	360	353	713
4	Climate	Differentiated	343	360	703
			1,422	1,433	2,855

Supplementary Table 3: Number of participants per experimental group.

3 Robustness checks

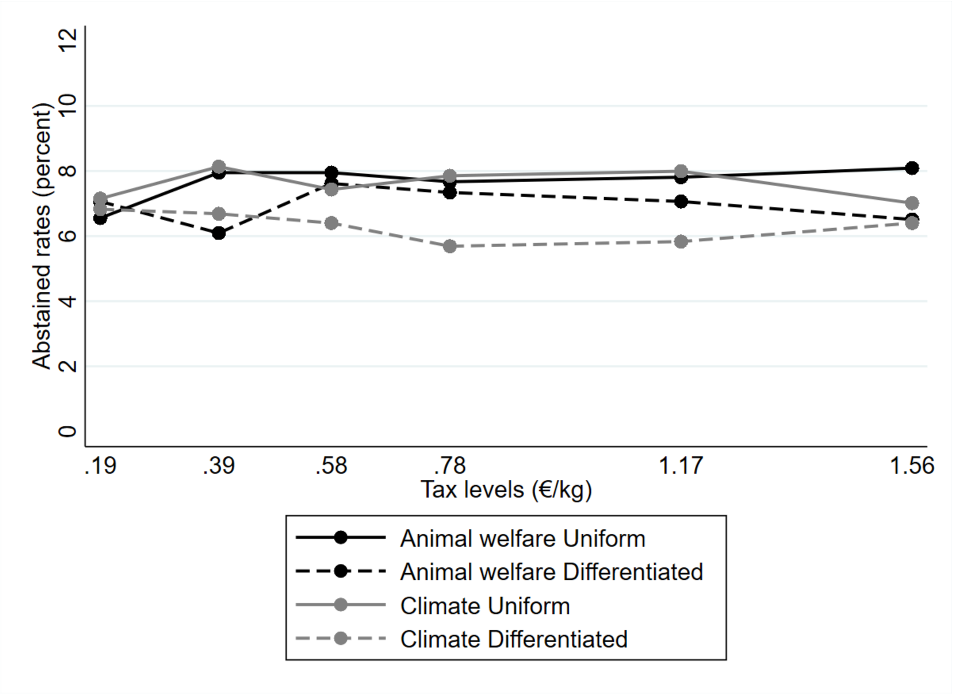

Supplementary Figure 4: Rates of abstention from voting across tax schemes. The graph shows the percentage of participants who chose `I do not want to vote.` at each tax level proposed by tax scheme. Tax justifications are distinguished by line color (animal welfare vs. climate) and degree of differentiation by line style (uniform vs. differentiated). The lowest tax level corresponds to a carbon price of €25/t CO_2, the highest tax level to €200/t CO_2

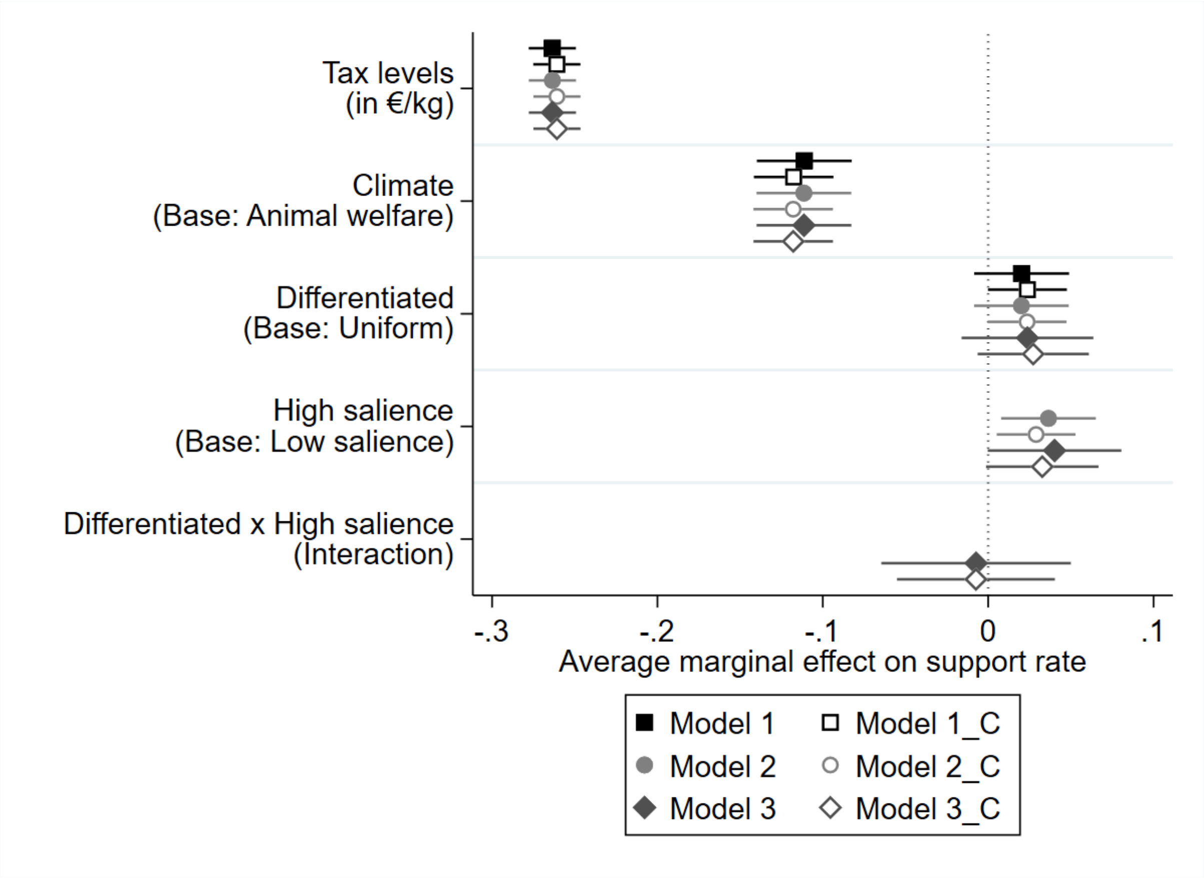

Supplementary Figure 5: Effect of tax attributes and salience as well as control variables on support for tax on meat. Data points indicate mean percentage point estimates with cluster-robust 95% confidence intervals from linear regressions of valid votes for a proposed tax scheme (1 for `yes`, 0 for `no`) for n=15,908 observations (corresponding to 2,759 respondents) in Models 1-3 and n=15,835 observations (corresponding to 2,745 respondents) in Models 1_C-3_C. Independent variables are tax levels in €/kg (continuous), tax justification (dummy variable: 0 for animal welfare, 1 for climate), degree of differentiation (dummy variable: 0 for uniform, 1 for differentiated tax), salience (dummy variable: 0 for low salience/belief elicitation task after referendum task, 1 for high salience/belief elicitation task before referendum task). Model 1 comprises independent variables tax levels, tax justification and degree of differentiation. In Model 2, salience is added. Model 3 comprises all independent variables mentioned, including the interaction term between degree of differentiation and salience. Models 1_C-3_C include the following control variables: demographics as shown in Supplementary Table 2, views on government, political preferences, voting for Green party, identifying as pescetarian/vegetarian/vegan, consuming meat, buying meat, purchase frequencies by meat type, purchase frequency of organic meat, importance of animal welfare/climate/organic in purchases, consequentiality perception, social desirability bias and attention.

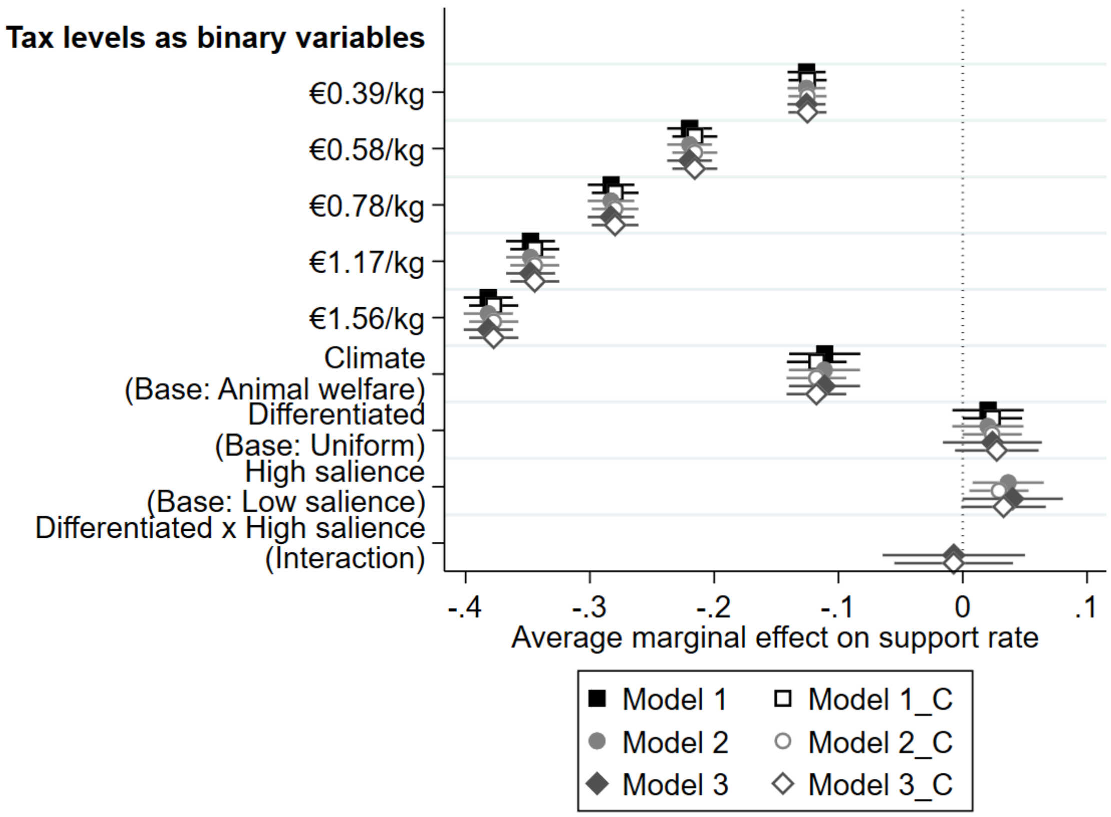

Supplementary Figure 6: Effect of tax levels as six binary variables, tax attributes and salience on support for tax on meat. Data points indicate mean percentage point estimates with cluster-robust 95% confidence intervals from linear regressions of valid votes for a proposed tax scheme (1 for `yes`, 0 for `no`) for n=15,908 observations (corresponding to 2,759 respondents) in Models 1-3 and n=15,835 observations (corresponding to 2,745 respondents) in Models 1_C-3_C. Independent variables are six tax levels in €/kg (6 dummy variables with €0.19/kg as the base category), tax justification (dummy variable: 0 for animal welfare, 1 for climate), degree of differentiation (dummy variable: 0 for uniform, 1 for differentiated tax) and salience (dummy variable: 0 for low salience/belief elicitation task after referendum task, 1 for high salience/belief elicitation task before referendum task). Model 1 comprises independent variables tax levels as six binary variables, tax justification and degree of differentiation. In Model 2, salience is added. Model 3 comprises all independent variables mentioned, including the interaction term between degree of differentiation and salience. Models 1_C-3_C include the following control variables: demographics as shown in Supplementary Table 2, views on government, political preferences, voting for Green party, identifying as pescetarian/vegetarian/vegan, consuming meat, buying meat, purchase frequencies by meat type, purchase frequency of organic meat, importance of animal welfare/climate/organic in purchases, consequentiality perception, social desirability bias and attention.

4 Regression tables

	(1)	(2)<br>Model 1	(3)<br>Model 1_C	(4)<br>Model 2	(5)<br>Model 2_C	(6)<br>Model 3	(7)<br>Model 3_C
Tax levels (in €/kg)	-0.264***<br>(0.007)	-0.264***<br>(0.007)	-0.261***<br>(0.007)	-0.264***<br>(0.007)	-0.261***<br>(0.007)	-0.264***<br>(0.007)	-0.261***<br>(0.007)
Climate		-0.111***<br>(0.015)	-0.118***<br>(0.012)	-0.111***<br>(0.015)	-0.118***<br>(0.012)	-0.111***<br>(0.015)	-0.118***<br>(0.012)
Differentiated tax		0.020<br>(0.015)	0.024*<br>(0.012)	0.020<br>(0.015)	0.024*<br>(0.012)	0.024<br>(0.020)	0.027<br>(0.017)
High salience				0.036**<br>(0.015)	0.029**<br>(0.012)	0.040*<br>(0.021)	0.033*<br>(0.017)
Differentiated x high salience						-0.007<br>(0.029)	-0.007<br>(0.024)
Constant	0.594***<br>(0.010)	0.639***<br>(0.014)	0.240***<br>(0.076)	0.621***<br>(0.016)	0.226***<br>(0.076)	0.619***<br>(0.017)	0.224***<br>(0.076)
Observations	15,908	15,908	15,835	15,908	15,835	15,908	15,835
Respondents	2,759	2,759	2,745	2,759	2,745	2,759	2,745
R^2	0.064	0.077	0.273	0.078	0.274	0.078	0.274
F-statistic	1,314.414	476.188	81.627	361.834	80.282	289.681	78.862

Supplementary Table 4: Linear regression table: Effect of tax attributes and salience on support for tax on meat. The table shows coefficient estimates from linear regressions of valid votes for a proposed tax scheme (1 for `yes`, 0 for `no`). Independent variables are tax levels in €/kg (continuous), tax justification (dummy variable: 0 for animal welfare, 1 for climate), degree of differentiation (dummy variable: 0 for uniform, 1 for differentiated tax) and salience (dummy variable: 0 for low salience/belief elicitation task after referendum task, 1 for high salience/belief elicitation task before referendum task). Models 1_C-3_C include the following control variables: demographics as shown in Supplementary Table 2, views on government, political preferences, voting for Green party, identifying as pescetarian/vegetarian/vegan, consuming meat, buying meat, purchase frequencies by meat type, purchase frequency of organic meat, importance of animal welfare/climate/organic in purchases, consequentiality perception, social desirability bias and attention. Abstentions from voting are excluded as observations. Observations for Models 1_C-3_C are lower because 15 participants chose not to report if they have kids. Robust standard errors, clustered on respondent level, in parentheses. Statistical tests are two-sided t-tests. No adjustments made for multiple comparisons. *: Significant at 10%; **: 5%; ***: 1%.

	(1)	(2) Model 1	(3) Model 1_C	(4) Model 2	(5) Model 2_C	(6) Model 3	(7) Model 3_C
€0.39/kg	-0.126***	-0.126***	-0.125***	-0.126***	-0.125***	-0.126***	-0.125***
	(0.008)	(0.008)	(0.008)	(0.008)	(0.008)	(0.008)	(0.008)
€0.58/kg	-0.220***	-0.220***	-0.216***	-0.220***	-0.216***	-0.220***	-0.216***
	(0.009)	(0.009)	(0.009)	(0.009)	(0.009)	(0.009)	(0.009)
€0.78/kg	-0.283***	-0.283***	-0.280***	-0.283***	-0.280***	-0.283***	-0.280***
	(0.010)	(0.010)	(0.010)	(0.010)	(0.010)	(0.010)	(0.010)
€1.17/kg	-0.348***	-0.348***	-0.344***	-0.348***	-0.344***	-0.348***	-0.344***
	(0.010)	(0.010)	(0.010)	(0.010)	(0.010)	(0.010)	(0.010)
€1.56/kg	-0.382***	-0.382***	-0.377***	-0.382***	-0.377***	-0.382***	-0.377***
	(0.010)	(0.010)	(0.010)	(0.010)	(0.010)	(0.010)	(0.010)
Climate		-0.111***	-0.118***	-0.111***	-0.118***	-0.111***	-0.118***
		(0.015)	(0.012)	(0.015)	(0.012)	(0.015)	(0.012)
Differentiated tax		0.020	0.024*	0.020	0.024*	0.024	0.027
		(0.015)	(0.012)	(0.015)	(0.012)	(0.020)	(0.017)
High salience				0.037**	0.029**	0.040*	0.033*
				(0.015)	(0.012)	(0.021)	(0.017)
Differentiated x high salience						-0.007	-0.007
						(0.029)	(0.024)
Constant	0.615***	0.660***	0.260***	0.642***	0.246***	0.640***	0.244***
	(0.009)	(0.014)	(0.076)	(0.016)	(0.076)	(0.017)	(0.077)
Observations	15,908	15,908	15,835	15,908	15,835	15,908	15,835
Respondents	2,759	2,759	2,745	2,759	2,745	2,759	2,745
R^2	0.073	0.086	0.282	0.088	0.283	0.088	0.283
F-statistic	291.470	225.576	80.625	199.642	79.358	177.601	78.058

Supplementary Table 5: Linear regression table: Effect of tax levels as six binary variables, tax attributes and salience on support for tax on meat. The table shows coefficient estimates from linear regressions of votes for a proposed tax scheme (1 for `yes`, 0 for `no`). Independent variables are six tax levels in €/kg (six dummy variables with €0.19/kg as the base category), tax justification (dummy variable: 0 for animal welfare, 1 for climate), degree of differentiation (dummy variable: 0 for uniform, 1 for differentiated tax) and salience (dummy variable: 0 for low salience/belief elicitation task after referendum task, 1 for high salience/belief elicitation task before referendum task). Models 1_C-3_C include all control variables as in Supplementary Table 4. Abstentions from voting are excluded as observations. Observations for Models 1_C-3_C are lower because 15 participants chose not to report if they have kids. Robust standard errors, clustered on respondent level, in parentheses. Statistical tests are two-sided t-tests. No adjustments made for multiple comparisons. *: Significant at 10%; **: 5%; ***: 1%

	(1) Model 1	(2) Model 1_C	(3) Model 2	(4) Model 2_C	(5) Model 3	(6) Model 3_C
**Part A: Average marginal effects**						
Tax levels (in €/kg)	-0.266***	-0.263***	-0.266***	-0.263***	-0.263***	-0.263***
	(0.007)	(0.007)	(0.007)	(0.007)	(0.007)	(0.007)
Climate	-0.111***	-0.118***	-0.111***	-0.119***	-0.119***	-0.119***
	(0.015)	(0.012)	(0.015)	(0.012)	(0.012)	(0.012)
Differentiated tax	0.020	0.023*	0.020	0.024*		
	(0.015)	(0.012)	(0.015)	(0.012)		
High salience			0.036**	0.028**	0.028**	0.028**
			(0.015)	(0.012)	(0.012)	(0.012)
For Differentiated:						
Low salience					0.024	0.026
					(0.020)	(0.017)
High salience					0.017	0.021
					(0.021)	(0.017)
**Part B: Odds ratios**						
Tax levels (in €/kg)	0.297***	0.212***	0.297***	0.211***	0.297***	0.211***
	(0.011)	(0.011)	(0.011)	(0.011)	(0.011)	(0.011)
Climate	0.604***	0.499***	0.603***	0.498***	0.603***	0.498***
	(0.040)	(0.036)	(0.040)	(0.036)	(0.040)	(0.036)
Differentiated tax	1.097	1.148*	1.096	1.149*	1.117	1.167
	(0.073)	(0.082)	(0.073)	(0.082)	(0.105)	(0.119)
High salience			1.181**	1.182**	1.203*	1.199*
			(0.079)	(0.084)	(0.114)	(0.123)
Differentiated x high salience					0.964	0.971
					(0.129)	(0.138)
Observations	15,908	15,835	15,908	15,835	15,908	15,835
Respondents	2,759	2,745	2,759	2,745	2,759	2,745
Pseudo R^2	0.060	0.238	0.061	0.239	0.061	0.239
Log pseudolikelihood	-9988.180	-8062.168	-9976.091	-8053.009	-9975.949	-8052.939

Supplementary Table 6: Logistic regression table: Effect of tax attributes and salience on support for tax on meat. For each model, the table shows average marginal effect estimates in (Part A) and odds ratio estimates (Part B) from logistic regressions of valid votes for a proposed tax scheme (1 for `yes`, 0 for `no`). Independent variables are tax levels in €/kg (continuous), tax justification (dummy variable: 0 for animal welfare, 1 for climate), degree of differentiation (dummy variable: 0 for uniform, 1 for differentiated tax) and salience (dummy variable: 0 for low salience/belief elicitation task after referendum task, 1 for high salience/belief elicitation task before referendum task). Models 1_C-3_C include all control variables as in Supplementary Table 4. Abstentions from voting are excluded as observations. Observations for Models 1_C-3_C are lower because 15 participants chose not to report if they have kids. Robust standard errors, clustered on respondent level, in parentheses. *: Significant at 10%; **: 5%; ***: 1%.

	Restricted sample		Unrestricted sample	
	(1)	(2)	(3)	(4)
Tax levels (in €/kg)	-0.264***	-0.261***	-0.256***	-0.254***
	(0.007)	(0.007)	(0.007)	(0.007)
Climate	-0.111***	-0.118***	-0.102***	-0.109***
	(0.015)	(0.012)	(0.014)	(0.012)
Differentiated tax	0.024	0.027	0.026	0.027
	(0.020)	(0.017)	(0.019)	(0.016)
High salience	0.040*	0.033*	0.037*	0.029*
	(0.021)	(0.017)	(0.020)	(0.017)
Differentiated x high salience	-0.007	-0.007	-0.009	-0.011
	(0.029)	(0.024)	(0.028)	(0.023)
Constant	0.619***	0.224***	0.617***	0.322***
	(0.017)	(0.076)	(0.017)	(0.065)
Observations	15,908	15,835	17,587	17,504
Respondents	2,759	2,745	3,057	3,041
R^2	0.078	0.274	0.072	0.264
F-statistic	289.681	78.862	300.379	81.901

Supplementary Table 7: Linear regression table: Effect of tax attributes and salience on support for tax on meat in restricted and unrestricted sample. The table shows coefficient estimates from linear regressions of valid votes for a proposed tax scheme (1 for `yes`, 0 for `no`) with both the restricted and unrestricted sample. (3) and (4) include respondents that took less (more) than 5 (45) minutes to complete the survey (equivalent to <5th and >95th percentile respectively). (1) and (2) are equal to Models 3 and 3_C in Supplementary Table 4. Independent variables are tax levels in €/kg (continuous), tax justification (dummy variable: 0 for animal welfare, 1 for climate), degree of differentiation (dummy variable: 0 for uniform, 1 for differentiated tax) and salience (dummy variable: 0 for low salience/belief elicitation task after referendum task, 1 for high salience/belief elicitation task before referendum task). (2) and (4) include control variables as in Supplementary Table 4. Abstentions from voting are excluded as observations. Observations for (2) and (4) are lower because 15 participants chose not to report if they have kids. Robust standard errors, clustered on respondent level, in parentheses. Statistical tests are two-sided t-tests. No adjustments made for multiple comparisons. *: Significant at 10%; **: 5%; ***: 1%.

	Decrease	Remain the same	Increase
**Overall meat consumption**			
Climate	0.019	-0.015	-0.004
	(0.018)	(0.014)	(0.004)
Differentiated tax	0.026	-0.021	-0.006
	(0.018)	(0.014)	(0.004)
High salience	0.021	-0.016	-0.004
	(0.018)	(0.014)	(0.004)
**Beef/Level 1**			
Climate	0.058***	0.022	-0.081***
	(0.018)	(0.017)	(0.011)
Differentiated tax	0.143***	-0.091***	-0.053***
	(0.018)	(0.012)	(0.007)
High salience	0.037**	-0.023**	-0.014**
	(0.018)	(0.011)	(0.007)
**Lamb/Level 2**			
Climate	0.142***	0.021	-0.163***
	(0.018)	(0.018)	(0.013)
Differentiated tax	0.064***	-0.032***	-0.032***
	(0.017)	(0.009)	(0.009)
High salience	0.024	-0.012	-0.012
	(0.017)	(0.009)	(0.009)
**Pork/Level 3**			
Climate	0.217***	0.109***	-0.326***
	(0.017)	(0.018)	(0.016)
Differentiated tax	-0.121***	0.009***	0.113***
	(0.014)	(0.003)	(0.013)
High salience	-0.015	0.001	0.013
	(0.014)	(0.001)	(0.013)
**Poultry/Level 4**			
Climate	0.051***	0.076***	-0.127***
	(0.016)	(0.018)	(0.018)
Differentiated tax	-0.134***	-0.045***	0.179***
	(0.012)	(0.006)	(0.016)
High salience	-0.019	-0.006	0.025
	(0.012)	(0.004)	(0.016)

Supplementary Table 8: Beliefs re. development of meat consumption in relation to tax on meat and tax attributes. The table shows estimates of average marginal effects from generalized ordered logistic regressions of beliefs re. development of meat consumption with answer levels (1) `Decrease`, (2) `Remain the same`, (3) `Increase`. Each sub-section represents results for a separate question on the development of consumption in the respective category. Independent variables are tax justification (dummy variable: 0 for animal welfare, 1 for climate), degree of differentiation (dummy variable: 0 for uniform, 1 for differentiated tax) and salience (dummy variable: 0 for low salience/belief elicitation task after referendum task, 1 for high salience/belief elicitation task before referendum task). Number of observations per question is 2,855. Statistical tests are two-sided z-tests. No adjustments made for multiple comparisons. Robust standard errors in parentheses. *: Significant at 10%; **: 5%; ***: 1%

	Overall meat consumption	Beef/Level 1	Lamb/Level 2	Pork/Level 3	Poultry/Level 4
**Decrease**					
Climate	0.927	0.780***	0.562***	0.341***	0.747***
	(0.068)	(0.060)	(0.043)	(0.029)	(0.067)
Differentiated tax	0.900	0.546***	0.769***	1.845***	2.155***
	(0.066)	(0.042)	(0.055)	(0.131)	(0.153)
High salience	0.920	0.854**	0.905	1.077	1.117
	(0.068)	(0.064)	(0.065)	(0.077)	(0.079)
**Remain the same**					
Climate	0.927	0.387***	0.245***	0.183***	0.576***
	(0.068)	(0.053)	(0.030)	(0.017)	(0.045)
Differentiated tax	0.900	0.546***	0.769***	1.845***	2.155***
	(0.066)	(0.042)	(0.055)	(0.131)	(0.153)
High salience	0.920	0.854**	0.905	1.077	1.117
	(0.068)	(0.064)	(0.065)	(0.077)	(0.079)
Observations	2,855	2,855	2,855	2,855	2,855
Log likelihood	-2,444.73	-2,508.56	-2,790.57	-2,881.04	-2,981.02
Pseudo R^2	0.001	0.024	0.031	0.076	0.028

Supplementary Table 9: Ordered logistic regression table: Beliefs re. development of meat consumption in relation to tax on meat and tax attributes. The table shows odds ratios from generalized ordered logistic regressions of beliefs re. development of meat consumption with answer levels (1) `Decrease`, (2) `Remain the same`, (3) `Increase`. Answer `Increase` is the base category. Each model represents results for a separate question on the development of consumption in the respective category. Independent variables are tax justification (dummy variable: 0 for animal welfare, 1 for climate), degree of differentiation (dummy variable: 0 for uniform, 1 for differentiated tax) and salience (dummy variable: 0 for low salience/belief elicitation task after referendum task, 1 for high salience/belief elicitation task before referendum task). Robust standard errors in parentheses. Statistical tests are two-sided z-tests. No adjustments made for multiple comparisons. *: Significant at 10%; **: 5%; ***: 1%

	Decrease	Remain the same	Increase
**Overall meat consumption**			
Climate	0.025	-0.020	-0.005
	(0.026)	(0.020)	(0.005)
High salience	0.041	-0.032	-0.009
	(0.026)	(0.020)	(0.005)
**Beef/Level 1**			
Climate	0.090***	0.023	-0.113***
	(0.026)	(0.025)	(0.017)
High salience	0.126***	-0.073***	-0.053***
	(0.025)	(0.015)	(0.011)
**Lamb/Level 2**			
Climate	0.174***	-0.006	-0.168***
	(0.026)	(0.026)	(0.019)
High salience	0.082***	-0.038***	-0.044***
	(0.024)	(0.011)	(0.013)
**Pork/Level 3**			
Climate	0.248***	0.077***	-0.325***
	(0.025)	(0.025)	(0.021)
High salience	-0.050**	0.012**	0.037**
	(0.022)	(0.006)	(0.016)
**Poultry/Level 4**			
Climate	0.095***	0.056**	-0.151***
	(0.024)	(0.026)	(0.024)
High salience	-0.049**	-0.000	0.050**
	(0.021)	(0.002)	(0.021)

Supplementary Table 10: Beliefs re. development of meat consumption in relation to tax on meat and tax attributes - Uniform tax only. The table shows estimates of average marginal effects from generalized ordered logistic regressions of beliefs re. development of meat consumption with answer levels (1) `Decrease`, (2) `Remain the same`, (3) `Increase`. Only participants who saw a uniform meat tax are considered. Each sub-section represents results for a separate question on the development of consumption in the respective category. Independent variables are tax justification (dummy variable: 0 for animal welfare, 1 for climate) and salience (dummy variable: 0 for low salience/belief elicitation task after referendum task, 1 for high salience/belief elicitation task before referendum task). Number of observations per question is 1,430. Robust standard errors in parentheses. Statistical tests are two-sided z-tests. No adjustments made for multiple comparisons. *: Significant at 10%; **: 5%; ***: 1%

	Overall meat consumption	Beef/Level 1	Lamb/Level 2	Pork/Level 3	Poultry/Level 4
**Decrease**					
Climate	0.905	0.692***	0.485***	0.336***	0.638***
	(0.094)	(0.074)	(0.053)	(0.038)	(0.074)
High salience	0.848	0.600***	0.708***	1.256**	1.266**
	(0.088)	(0.062)	(0.072)	(0.126)	(0.124)
**Remain the same**					
Climate	0.905	0.321***	0.255***	0.144***	0.492***
	(0.094)	(0.058)	(0.042)	(0.021)	(0.057)
High salience	0.848	0.600***	0.708***	1.256**	1.266**
	(0.088)	(0.062)	(0.072)	(0.126)	(0.124)
Observations	1,430	1,430	1,430	1,430	1,430
Log likelihood	-1,231.46	-1,343.27	-1,411.60	-1,436.34	-1,541.94
Pseudo R^2	0.001	0.025	0.035	0.074	0.015

Supplementary Table 11: Ordered logistic regression table: Beliefs re. development of meat consumption in relation to tax on meat and tax attributes - Uniform tax only. The table shows odds ratios from generalized ordered logistic regressions of beliefs re. development of meat consumption with answer levels (1) `Decrease`, (2) `Remain the same`, (3) `Increase`. Answer `Increase` is the base category. Only participants who saw a uniform meat tax are considered. Each model represents results for a separate question on the development of consumption in the respective category. Independent variables are tax justification (dummy variable: 0 for animal welfare, 1 for climate) and salience (dummy variable: 0 for low salience/belief elicitation task after referendum task, 1 for high salience/belief elicitation task before referendum task). Robust standard errors in parentheses. Statistical tests are two-sided z-tests. No adjustments made for multiple comparisons. *: Significant at 10%; **: 5%; ***: 1%

5 Survey questionnaire

The survey was conducted in German. The questionnaires on the following pages have been translated into English.

Pages 19-20 show the quota questionnaire. Quotas regarding sex, age, region of living, education and monthly net household income were tracked by the panel provider. The first five questions were implemented on its website. Respondents who matched the quota were redirected to our survey questionnaire, starting on page 21.

The term *QUOTA* on page 34 refers to the experimental groups. At this point in the survey, respondents are randomized into one of the eight experimental groups.

The survey questionnaire contains placeholder elements (*Script*) due to the programming structure of the survey. The placeholder is replaced by information on the respective tax scheme depending on the experimental group respondents have been randomized into. Starting on page 71, we added screenshots of how these placeholders looked like in the survey.

IntroSurvev:

Welcome to the study on eating habits

This is a scientific study conducted on behalf of the University of Hamburg. It will take about **15 minutes** to answer the questions. In the course of the survey, you have the chance to receive an additional bonus in the amount of max. 100 Mingle points. The corresponding questions are marked with the heading "Bonus“.

Please answer all questions as conscientiously and honestly as possible. All information will be treated confidentially and is anonymous. It will not be possible to link your answers to your name or address. The information will only be used for scientific purposes and under no circumstances will personal data be passed on to third parties.

Please feel free to submit comments on this survey after it is completed.

Thank you for your support!

---------------------------------<PAGE BREAK>----------------------------------

HARD QUOTAS

age:

Please indicate your age (in years).

[ ] years

Post-Skip: Screen out if age<18

sex:

Please indicate your sex.

1 Female

2 Male

region:

Please indicate in which federal state you live.

1 Baden-Württemberg

2 Bayern

3 Berlin

4 Brandenburg

5 Bremen

6 Hamburg

7 Hessen

8 Mecklenburg-Vorpommern

9 Niedersachsen

10 Nordrhein-Westfalen

11 Rheinland-Pfalz

12 Saarland

13 Sachsen

14 Sachsen-Anhalt

15 Schleswig-Holstein

16 Thüringen

----------------------------------<PAGE BREAK>----------------------------------

SOFT QUOTAS

income:

What is the total **net monthly income of your household**?
(Sum remaining after deduction of taxes and social security contributions)

1 Under 1,000 Euro

2 1,000 - under 2,000 Euro

3 2,000 - under 4,000 Euro

4 4,000 – 6,000 Euro

5 Over 6,000 Euro

----------------------------------<PAGE BREAK>----------------------------------

education:

What is the highest level of education you have attained?

1 Still in school/no school-leaving qualification

2 Certificate of general secondary education

3 Certificate of intermediate secondary education

4 University-entrance diploma

5 University degree/PhD

0% 100%

memhouse

How many people live permanently in your household, including yourself?
Your household includes all persons who live there together. Please also consider all children living in the household.

[ ] Adults

[ ] Minors (under 18 years)

kids

Do you have children?
Please also think about your adult children and children who do not (no longer) live in your household.

- Yes
- No
- Not specified

Next

0% 100%

job

What is your current job status?

- ◯ In training (school, vocational training, study)
- ◯ Parental leave
- ◯ Housewife/-man
- ◯ Employed full time
- ◯ Employed part time
- ◯ Self-employed/freelance
- ◯ Pension/retirement/early retirement
- ◯ Jobseeker/unemployed
- ◯ Leave without pay/sabbatical
- ◯ Other *(Please specify)* ____________

Next

0% 100%

media

There are different sources from which you can get information about news and latest events. Where do you get the most information about news and latest events?

Multiple selection possible

- ☐ Newspapers/magazines (printed and/or online)
- ☐ Social media (Facebook, Twitter, Instagram)
- ☐ Websites on the Internet
- ☐ TV
- ☐ Radio
- ☐ Other sources ____________
- ☐ Not specified

Next

0% 100%

govinv

Some people feel that the government takes on too many tasks that should be left to individuals and businesses. Others think the government should do more to solve the country's problems.
What is your assessment?

The government does...

way too much	too much	a little too much	neither too much nor too little	a bit too little	too little	way too little	Not specified
◯	◯	◯	◯	◯	◯	◯	◯

govtrust

How much trust do you have in the German government in general?

Very low	Low	Rather low	Neither	Rather high	High	Very high	Not specified
◯	◯	◯	◯	◯	◯	◯	◯

Next

0% 100%

polview

In political discussions, people often talk about "left" and "right".
Where do you classify your own political position?

Left	Rather left	Center-left	Center	Center-right	Rather right	Right	Not specified
○	○	○	○	○	○	○	○

Next

0% 100%

polpo

Which party would you vote for if there were a federal election next Sunday?

- ◯ CDU/CSU
- ◯ SPD
- ◯ FDP
- ◯ Bündnis 90/Grüne
- ◯ Die Linke
- ◯ AfD
- ◯ Other party [ ]
- ◯ I would not go to the polls
- ◯ I am not eligible to vote
- ◯ Don't know
- ◯ Not specified

Next

0% 100%

conhab

Which of the following descriptions of dietary behavior applies to you?

I eat...

Please select all that apply.

- [ ] gluten-free (avoidance of wheat flour, etc.)
- [ ] lactose-free (no lactose)
- [ ] flexitarian (predominantly abstaining from meat, fish and sausages)
- [ ] pescetarian (abstain from meat and sausages, but not fish)
- [ ] vegetarian (avoidance of meat, fish and sausages)
- [ ] vegan (renunciation of all animal products)
- [ ] halal (adherence to the dietary laws of Islam)
- [ ] kosher (adherence to the dietary laws of Judaism)
- [ ] non-alcoholic
- [ ] others ______
- [ ] None of the above descriptions apply.

Next

0% 100%

meatpur

How often do you usually **purchase** the following types of meat for yourself and/or members of your household?

Please also consider sausages, cold cuts or prepared meat dishes that contain these meats.

	Several times a week	Once a week	Several times a month	Once a month	Less than once a month	Never
Beef, veal	◯	◯	◯	◯	◯	◯
Pork	◯	◯	◯	◯	◯	◯
Poultry (including chicken, turkey, goose, duck)	◯	◯	◯	◯	◯	◯
Lamb, sheep, goat	◯	◯	◯	◯	◯	◯
Others (e.g., venison, etc.)	◯	◯	◯	◯	◯	◯

Next

0% 100%

pop

Where do you purchase meat, including sausages, cold cuts or prepared meat dishes?

Please select each location that applies.

- ☐ Supermarket
- ☐ Discounter
- ☐ (Weekly) Market
- ☐ Internet
- ☐ Specialty store (e.g. butcher, delicatessen)
- ☐ Organic shop/Reformhaus
- ☐ Directly from the farmer/farm store
- ☐ Gas station
- ☐ In restaurant/snack bar/take-away etc.
- ☐ Others ______

organiccons

If you generally buy meat, how often do you buy certified organic meat products, including sausages or cold cuts?

Never	Rarely	Rather rarely	Sometimes	Rather often	Often	Always
○	○	○	○	○	○	○

Next

0% 100%

meatcon

How often do you usually **eat** the following types of meat?

Please also consider sausages, cold cuts or prepared meat dishes that contain these meats.

	Several times a day	Once a day	Several times a week	Once a week	Several times a month	Once a month	Less than once a month	Never
Beef, veal	◯	◯	◯	◯	◯	◯	◯	◯
Pork	◯	◯	◯	◯	◯	◯	◯	◯
Poultry (including chicken, turkey, goose, duck)	◯	◯	◯	◯	◯	◯	◯	◯
Lamb, sheep, goat	◯	◯	◯	◯	◯	◯	◯	◯
Others (e.g., venison, etc.)	◯	◯	◯	◯	◯	◯	◯	◯

Next

0% 100%

screen1

Recent research on decision making shows that decisions are influenced by context. Different human emotions, prior knowledge, experience and the environment can influence decisions. To understand how people make decisions, we are interested in your assessment. In particular, we are interested in whether you have taken the time to read the question to this point. If not, some of your answers tell us little about how you make decisions in reality. To show that you have read this far, please ignore the question below about your feelings. Instead, please select "None of the above" as your answer.

Please choose any words that describe how you are feeling right now.

☐ Proud	☐ Excited	☐ Active
☐ Interested	☐ Stressed	☐ Determined
☐ Frightened	☐ Nervous	☐ Restless
☐ Anxious	☐ Strong	☐ Enthusiastic
☐ Angry	☐ Attentive	☐ None of the above

Next

0% 100%

diff

How difficult would it be for you to ...

	Very difficult	Difficult	Relatively difficult	Neither difficult nor easy	Relatively easy	Easy	Very easy
reduce your consumption of meat products?	◯	◯	◯	◯	◯	◯	◯
give up meat products completely?	◯	◯	◯	◯	◯	◯	◯

Next

0% 100%

shopimp

How important are the following aspects to you when you shop for groceries?

	Not important at all	Unimportant	Rather unimportant	Neither important nor unimportant	Rather important	Important	Very important
Health benefits and nutritional values	○	○	○	○	○	○	○
Fair Trade	○	○	○	○	○	○	○
Animal welfare	○	○	○	○	○	○	○
Taste	○	○	○	○	○	○	○
Food safety	○	○	○	○	○	○	○
Shelf life	○	○	○	○	○	○	○
Brand	○	○	○	○	○	○	○
Convenience	○	○	○	○	○	○	○
Price	○	○	○	○	○	○	○
Climate and environmental impact	○	○	○	○	○	○	○
Organic certified	○	○	○	○	○	○	○
Freshness	○	○	○	○	○	○	○
Regional	○	○	○	○	○	○	○

Next

0% 100%

quota

[QUOTA: quota]

com

The following pages are about your opinion on the introduction of a **levy on meat products** in Germany. You will receive some information about this in a moment.

Please note: With this survey, we would like to capture as accurately as possible the opinion of the German population on this topic. It is therefore **very important** that you **read all the information provided carefully** and **answer the questions honestly**.

Do you agree to **read** the information **carefully** and answer the questions **honestly**?

- ◯ Yes, I will read carefully and give honest answers.
- ◯ No. I will not read carefully and not give honest answers.
- ◯ I can promise neither one nor the other.

Next

0% 100%

extax

Many measures on meat consumption are currently being discussed in German politics.

One proposal is to impose an **additional levy on meat products** (fresh meat, sausages and cold cuts).
Please read the following proposal carefully.

[Script]

clicked

Next

0% 100%

exref

Now imagine that you can **participate in a Germany-wide referendum** on the [Script] shown on the previous page.

On the next pages you will see **6 different proposals** one after the other. All proposals differ only in the amount of the levy.

As in a real referendum, you can **vote for or against** the proposal shown.

Please consider each proposal independently of the others. Vote as if the **proposal shown were the only one on the ballot**.

Your responses are important and should be heard.

After completion of the survey, we will send the results of this part of the survey to the **Committee on Food and Agriculture** and the **Committee on the Environment, Nature Conservation and Nuclear Safety** of the **German Bundestag**.

For each of the 6 proposals you are about to see, we inform the committees of the **proportion of respondents who voted for or against**. You can see the template for the letter to the committees below.

The letter is really sent to the two committees mentioned.

We only communicate the summarized results of the vote to the committees. **Your personal vote remains anonymous and secret.** No one, including the research team, can match your answers to your name or identity.

Template for the letter:

[Script]

Next

0% 100%

clibelownall

[Script] How do you think **general meat consumption** (incl. sausages and cold cuts) in Germany will change if the previously shown climate levy on meat products is introduced?
Here you can see once again all the details of the levy.

General meat consumption...

will decrease ➘	will increase ➚	will remain the same ➙
○	○	○

clibelowndiff

How do you think consumption **by meat type** will change in Germany if the animal welfare levy on meat products shown earlier is introduced?

The consumption of...

	will decrease ➘	will increase ➚	will remain the same ➙
Beef	○	○	○
Lamb	○	○	○
Pork	○	○	○
Poultry	○	○	○

Next

0% 100%

clibelothallintro

This survey asks people between the ages of 18 and 74 who live in Germany and maps the actual population structure of Germany for this age group.

What do you think?
Out of 100 respondents who were also asked the question about the development of **general meat consumption**, how many gave the answers "will decrease" and "will increase"?

BONUS

Please estimate 2 numbers on this page: one for each answer *"will decrease"* and *"will increase"*. The value for the answer *"will remain the same"* is calculated automatically in each line from your entries.
For each guess, if your estimated number differs from the correct number by 2 or less, you will receive an additional payment of **10 mingle points**. So you can get an additional payment of **max. 20 mingle points** on this page.

clibelothall

How many of 100 respondents gave the following answers?

	will decrease ↘	will increase ↗
General meat consumption...		

The value for *"will remain the same"* must be at least 0 and must not be negative. The sum must add up to 100.

Next

0% 100%

clibelothdiffintro

We also asked you how consumption by meat type in Germany would develop as a result of the climate levy shown.

What do you think?
Out of 100 respondents who were also asked the questions about the development of **meat consumption by meat type**, how many gave the answers "will decrease" and "will increase"?

BONUS

Please estimate 4x2 numbers on this page: two for each meat type for the answers *"will decrease"* and *"will increase"* . The value for the answer *"will remain the same"* is calculated automatically in each line from your entries.
For each guess, if your estimated number differs from the correct number by 2 or less, you will receive an additional payment of **10 mingle points**. So you can get an additional payment of **max. 80 mingle points** on this page.

clibelothdiff

How many of 100 respondents gave the following answers?

The consumption of...

	will decrease ↘	will increase ↗
Beef		
Lamb		
Pork		
Poultry		

The value for *"will remain the same"* must be at least 0 and must not be negative. The sum must add up to 100.

Next

0% 100%

awbelownall

[Script] How do you think **general meat consumption** (incl. sausages and cold cuts) in Germany will change if the previously shown animal welfare levy on meat products is introduced?
Here you can see once again all the details of the levy.

General meat consumption...

will decrease ➘	will increase ➚	will remain the same ➙
◯	◯	◯

awbelowndiff

How do you think consumption **by husbandry level** will change in Germany if the animal welfare levy on meat products shown earlier is introduced?

The consumption of...

	will decrease ➘	will increase ➚	will remain the same ➙
Level 1 (Stable housing)	◯	◯	◯
Level 2 (Stable housing Plus)	◯	◯	◯
Level 3 (Outside climate)	◯	◯	◯
Level 4 (Premium/Organic)	◯	◯	◯

Next

0% 100%

awbelothallintro

This survey asks people between the ages of 18 and 74 who live in Germany and maps the actual population structure of Germany for this age group.

What do you think?
Out of 100 respondents who were also asked the question about the development of **general meat consumption**, how many gave the answers "will decrease" and "will increase"?

BONUS

Please estimate 2 numbers on this page: one for each answer *"will decrease"* and *"will increase"* . The value for the answer *"will remain the same"* is calculated automatically in each line from your entries.
For each guess, if your estimated number differs from the correct number by 2 or less, you will receive an additional payment of **10 mingle points**. So you can get an additional payment of **max. 20 mingle points** on this page.

awbelothall

How many of 100 respondents gave the following answers?

	will decrease ↘	will increase ↗
General meat consumption...		

The value for *"will remain the same"* must be at least 0 and must not be negative. The sum must add up to 100.

Next

0% 100%

awbelothdiffintro

We also asked you how consumption by husbandry level would develop in Germany as a result of the animal welfare levy shown.

What do you think?
Out of 100 respondents who were also asked the questions about the development of **meat consumption by husbandry level**, how many gave the answers "will decrease" and "will increase"?

BONUS

Please estimate 4x2 numbers on this page: two for each husbandry level for the answers *"will decrease"* and *"will increase"* . The value for the answer *"will remain the same"* is calculated automatically in each line from your entries.
For each guess, if your estimated number differs from the correct number by 2 or less, you will receive an additional payment of **10 mingle points**. So you can get an additional payment of **max. 80 mingle points** on this page.

awbelothdiff

How many of 100 respondents gave the following answers?

The consumption of...

	will decrease ↘	will increase ↗
Level 1 (Stable housing)		
Level 2 (Stable housing Plus)		
Level 3 (Outside climate)		
Level 4 (Premium/Organic)		

The value for *"will remain the same"* must be at least 0 and must not be negative. The sum must add up to 100.

Next

0% 100%

cliflar1

Please vote now:

Please vote as if the shown proposal was **the only one available for vote**.

1. Proposal for a climate levy:

PROPOSAL FOR VOTING	
Additional levy on meat products	Yes – Per kilogramm meat
Justification for the levy	Greenhouse gas emissions of meat production
Usage of revenues from the levy	Investments in climate protection
**Amount of the levy**	**Identical** for all meat types, no levy for plant-based alternatives

	Levy by meat type
**Beef**	+0,19 EUR/kg
**Lamb**	+0,19 EUR/kg
**Pork**	+0,19 EUR/kg
**Poultry**	+0,19 EUR/kg
**Plant-based alternative**	+0,00 EUR/kg

If the majority of valid votes (>50%) is against this proposal, no [Script] will be implemented.

Do you vote for this proposal?

- Yes. I vote **for** the introduction of this levy.
- No. I vote **against** the introduction of this levy.
- I do not want to vote.

Next

0% 100%

cliflar2

Please vote as if the shown proposal was **the only one available for vote**.

2. Proposal for a climate levy:

PROPOSAL FOR VOTING	
Additional levy on meat products	Yes – Per kilogramm meat
Justification for the levy	Greenhouse gas emissions of meat production
Usage of revenues from the levy	Investments in climate protection
**Amount of the levy**	**Identical** for all meat types, no levy for plant-based alternatives

	**Levy by meat type**
**Beef**	+0,39 EUR/kg
**Lamb**	+0,39 EUR/kg
**Pork**	+0,39 EUR/kg
**Poultry**	+0,39 EUR/kg
**Plant-based alternative**	+0,00 EUR/kg

If the majority of valid votes (>50%) is against this proposal, no [Script] will be implemented.

Do you vote for this proposal?

- Yes. I vote **for** the introduction of this levy.
- No. I vote **against** the introduction of this levy.
- I do not want to vote.

Next

0% 100%

cliflar3

Please vote as if the shown proposal was **the only one available for vote**.

3. Proposal for a climate levy:

PROPOSAL FOR VOTING	
Additional levy on meat products	Yes – Per kilogramm meat
Justification for the levy	Greenhouse gas emissions of meat production
Usage of revenues from the levy	Investments in climate protection
**Amount of the levy**	**Identical** for all meat types, no levy for plant-based alternatives

	Levy by meat type
**Beef**	+0,58 EUR/kg
**Lamb**	+0,58 EUR/kg
**Pork**	+0,58 EUR/kg
**Poultry**	+0,58 EUR/kg
**Plant-based alternative**	+0,00 EUR/kg

If the majority of valid votes (>50%) is against this proposal, no [Script] will be implemented.

Do you vote for this proposal?

- Yes. I vote **for** the introduction of this levy.
- No. I vote **against** the introduction of this levy.
- I do not want to vote.

Next

0% 100%

cliflar4

Please vote as if the shown proposal was **the only one available for vote**.

4. Proposal for a climate levy:

PROPOSAL FOR VOTING	
Additional levy on meat products	Yes – Per kilogramm meat
Justification for the levy	Greenhouse gas emissions of meat production
Usage of revenues from the levy	Investments in climate protection
**Amount of the levy**	**Identical** for all meat types, no levy for plant-based alternatives

	Levy by meat type
**Beef**	+0,78 EUR/kg
**Lamb**	+0,78 EUR/kg
**Pork**	+0,78 EUR/kg
**Poultry**	+0,78 EUR/kg
**Plant-based alternative**	+0,00 EUR/kg

If the majority of valid votes (>50%) is against this proposal, no [Script] will be implemented.

Do you vote for this proposal?

◯ Yes. I vote **for** the introduction of this levy.

◯ No. I vote **against** the introduction of this levy.

◯ I do not want to vote.

Next

0% 100%

cliflar5

Please vote as if the shown proposal was **the only one available for vote**.

5. Proposal for a climate levy:

PROPOSAL FOR VOTING	
Additional levy on meat products	Yes – Per kilogramm meat
Justification for the levy	Greenhouse gas emissions of meat production
Usage of revenues from the levy	Investments in climate protection
**Amount of the levy**	**Identical** for all meat types, no levy for plant-based alternatives

	**Levy by meat type**
**Beef**	+1,17 EUR/kg
**Lamb**	+1,17 EUR/kg
**Pork**	+1,17 EUR/kg
**Poultry**	+1,17 EUR/kg
**Plant-based alternative**	+0,00 EUR/kg

If the majority of valid votes (>50%) is against this proposal, no [Script] will be implemented.

Do you vote for this proposal?

- Yes. I vote **for** the introduction of this levy.
- No. I vote **against** the introduction of this levy.
- I do not want to vote.

Next

0% 100%

cliflar6

Please vote as if the shown proposal was **the only one available for vote**.

6. Proposal for a climate levy:

PROPOSAL FOR VOTING	
Additional levy on meat products	Yes – Per kilogramm meat
Justification for the levy	Greenhouse gas emissions of meat production
Usage of revenues from the levy	Investments in climate protection
**Amount of the levy**	**Identical** for all meat types, no levy for plant-based alternatives

	Levy by meat type
**Beef**	+1,56 EUR/kg
**Lamb**	+1,56 EUR/kg
**Pork**	+1,56 EUR/kg
**Poultry**	+1,56 EUR/kg
**Plant-based alternative**	+0,00 EUR/kg

If the majority of valid votes (>50%) is against this proposal, no [Script] will be implemented.

Do you vote for this proposal?

- Yes. I vote **for** the introduction of this levy.
- No. I vote **against** the introduction of this levy.
- I do not want to vote.

Next

0% 100%

awflar1

Please vote now:

Please vote as if the shown proposal was **the only one available for vote**.

1. Proposal for an animal welfare levy:

PROPOSAL FOR VOTING	
Additional levy on meat products	Yes – Per kilogramm meat
Justification for the levy	Animal welfare in meat production
Usage of revenues from the levy	Investments in improvement of animal welfare in livestock farming
**Amount of the levy**	**Identical** for all husbandry levels, no levy for plant-based alternatives

	**Levy by husbandry level**
**Level 1 (Stable housing)**	+0,19 EUR/kg
**Level 2 (Stable housing Plus)**	+0,19 EUR/kg
**Level 3 (Outside climate)**	+0,19 EUR/kg
**Level 4 (Premium)**	+0,19 EUR/kg
**Plant-based alternative**	+0,00 EUR/kg

If the majority of valid votes (>50%) is against this proposal, no [Script] will be implemented.

Do you vote for this proposal?

- Yes. I vote **for** the introduction of this levy.
- No. I vote **against** the introduction of this levy.
- I do not want to vote.

Next

0% 100%

awflar2

Please vote as if the shown proposal was **the only one available for vote**.

2. Proposal for an animal welfare levy:

PROPOSAL FOR VOTING	
Additional levy on meat products	Yes – Per kilogramm meat
Justification for the levy	Animal welfare in meat production
Usage of revenues from the levy	Investments in improvement of animal welfare in livestock farming
**Amount of the levy**	**Identical** for all husbandry levels, no levy for plant-based alternatives

	**Levy by husbandry level**
**Level 1 (Stable housing)**	+0,39 EUR/kg
**Level 2 (Stable housing Plus)**	+0,39 EUR/kg
**Level 3 (Outside climate)**	+0,39 EUR/kg
**Level 4 (Premium)**	+0,39 EUR/kg
**Plant-based alternative**	+0,00 EUR/kg

If the majority of valid votes (>50%) is against this proposal, no [Script] will be implemented.

Do you vote for this proposal?

- Yes. I vote **for** the introduction of this levy.
- No. I vote **against** the introduction of this levy.
- I do not want to vote.

Next

0% 100%

awflar3

Please vote as if the shown proposal was **the only one available for vote**.

3. Proposal for an animal welfare levy:

PROPOSAL FOR VOTING	
Additional levy on meat products	Yes – Per kilogramm meat
Justification for the levy	Animal welfare in meat production
Usage of revenues from the levy	Investments in improvement of animal welfare in livestock farming
**Amount of the levy**	**Identical** for all husbandry levels, no levy for plant-based alternatives

	**Levy by husbandry level**
**Level 1 (Stable housing)**	+0,58 EUR/kg
**Level 2 (Stable housing Plus)**	+0,58 EUR/kg
**Level 3 (Outside climate)**	+0,58 EUR/kg
**Level 4 (Premium)**	+0,58 EUR/kg
**Plant-based alternative**	+0,00 EUR/kg

If the majority of valid votes (>50%) is against this proposal, no [Script] will be implemented.

Do you vote for this proposal?

- ◯ Yes. I vote **for** the introduction of this levy.
- ◯ No. I vote **against** the introduction of this levy.
- ◯ I do not want to vote.

Next

0% 100%

awflar4

Please vote as if the shown proposal was **the only one available for vote**.

4. Proposal for an animal welfare levy:

PROPOSAL FOR VOTING	
Additional levy on meat products	Yes – Per kilogramm meat
Justification for the levy	Animal welfare in meat production
Usage of revenues from the levy	Investments in improvement of animal welfare in livestock farming
**Amount of the levy**	**Identical** for all husbandry levels, no levy for plant-based alternatives

	**Levy by husbandry level**
**Level 1 (Stable housing)**	+0,78 EUR/kg
**Level 2 (Stable housing Plus)**	+0,78 EUR/kg
**Level 3 (Outside climate)**	+0,78 EUR/kg
**Level 4 (Premium)**	+0,78 EUR/kg
**Plant-based alternative**	+0,00 EUR/kg

If the majority of valid votes (>50%) is against this proposal, no [Script] will be implemented.

Do you vote for this proposal?

- ◯ Yes. I vote **for** the introduction of this levy.
- ◯ No. I vote **against** the introduction of this levy.
- ◯ I do not want to vote.

Next

0% 100%

awflar5

Please vote as if the shown proposal was **the only one available for vote**.

5. Proposal for an animal welfare levy:

PROPOSAL FOR VOTING	
Additional levy on meat products	Yes – Per kilogramm meat
Justification for the levy	Animal welfare in meat production
Usage of revenues from the levy	Investments in improvement of animal welfare in livestock farming
**Amount of the levy**	**Identical** for all husbandry levels, no levy for plant-based alternatives

	Levy by husbandry level
**Level 1 (Stable housing)**	+1,17 EUR/kg
**Level 2 (Stable housing Plus)**	+1,17 EUR/kg
**Level 3 (Outside climate)**	+1,17 EUR/kg
**Level 4 (Premium)**	+1,17 EUR/kg
**Plant-based alternative**	+0,00 EUR/kg

If the majority of valid votes (>50%) is against this proposal, no [Script] will be implemented.

Do you vote for this proposal?

- ◯ Yes. I vote **for** the introduction of this levy.
- ◯ No. I vote **against** the introduction of this levy.
- ◯ I do not want to vote.

Next

0% 100%

awflar6

Please vote as if the shown proposal was **the only one available for vote**.

6. Proposal for an animal welfare levy:

PROPOSAL FOR VOTING	
Additional levy on meat products	Yes – Per kilogramm meat
Justification for the levy	Animal welfare in meat production
Usage of revenues from the levy	Investments in improvement of animal welfare in livestock farming
**Amount of the levy**	**Identical** for all husbandry levels, no levy for plant-based alternatives

	Levy by husbandry level
**Level 1 (Stable housing)**	+1,56 EUR/kg
**Level 2 (Stable housing Plus)**	+1,56 EUR/kg
**Level 3 (Outside climate)**	+1,56 EUR/kg
**Level 4 (Premium)**	+1,56 EUR/kg
**Plant-based alternative**	+0,00 EUR/kg

If the majority of valid votes (>50%) is against this proposal, no [Script] will be implemented.

Do you vote for this proposal?

- ◯ Yes. I vote **for** the introduction of this levy.
- ◯ No. I vote **against** the introduction of this levy.
- ◯ I do not want to vote.

Next

0% 100%

awdiffr1

Please vote now:

Please vote as if the shown proposal was **the only one available for vote**.

1. Proposal for an animal welfare levy:

PROPOSAL FOR VOTING	
Additional levy on meat products	Yes – Per kilogramm meat
Justification for the levy	Animal welfare in meat production
Usage of revenues from the levy	Investments in improvement of animal welfare in livestock farming
**Amount of the levy**	**Dependent** on husbandry level, no levy for plant-based alternatives

	**Levy by husbandry level**
**Level 1 (Stable housing)**	+0,54 EUR/kg
**Level 2 (Stable housing Plus)**	+0,44 EUR/kg
**Level 3 (Outside climate)**	+0,14 EUR/kg
**Level 4 (Premium)**	+0,08 EUR/kg
**Plant-based alternative**	+0,00 EUR/kg

If the majority of valid votes (>50%) is against this proposal, no [Script] will be implemented.

Do you vote for this proposal?

- ◯ Yes. I vote **for** the introduction of this levy.
- ◯ No. I vote **against** the introduction of this levy.
- ◯ I do not want to vote.

Next

0% 100%

awdiffr2

Please vote as if the shown proposal was **the only one available for vote**.

2. Proposal for an animal welfare levy:

PROPOSAL FOR VOTING	
Additional levy on meat products	Yes – Per kilogramm meat
Justification for the levy	Animal welfare in meat production
Usage of revenues from the levy	Investments in improvement of animal welfare in livestock farming
**Amount of the levy**	**Dependent** on husbandry level, no levy for plant-based alternatives

	**Levy by husbandry level**
**Level 1 (Stable housing)**	+1,08 EUR/kg
**Level 2 (Stable housing Plus)**	+0,88 EUR/kg
**Level 3 (Outside climate)**	+0,27 EUR/kg
**Level 4 (Premium)**	+0,16 EUR/kg
**Plant-based alternative**	+0,00 EUR/kg

If the majority of valid votes (>50%) is against this proposal, no [Script] will be implemented.

Do you vote for this proposal?

- Yes. I vote **for** the introduction of this levy.
- No. I vote **against** the introduction of this levy.
- I do not want to vote.

Next

0% 100%

awdiffr3

Please vote as if the shown proposal was **the only one available for vote**.

3. Proposal for an animal welfare levy:

PROPOSAL FOR VOTING	
Additional levy on meat products	Yes – Per kilogramm meat
Justification for the levy	Animal welfare in meat production
Usage of revenues from the levy	Investments in improvement of animal welfare in livestock farming
**Amount of the levy**	**Dependent** on husbandry level, no levy for plant-based alternatives

	**Levy by husbandry level**
**Level 1 (Stable housing)**	+1,61 EUR/kg
**Level 2 (Stable housing Plus)**	+1,32 EUR/kg
**Level 3 (Outside climate)**	+0,41 EUR/kg
**Level 4 (Premium)**	+0,23 EUR/kg
**Plant-based alternative**	+0,00 EUR/kg

If the majority of valid votes (>50%) is against this proposal, no [Script] will be implemented.

Do you vote for this proposal?

- Yes. I vote **for** the introduction of this levy.
- No. I vote **against** the introduction of this levy.
- I do not want to vote.

Next

0% 100%

awdiffr4

Please vote as if the shown proposal was **the only one available for vote**.

4. Proposal for an animal welfare levy:

PROPOSAL FOR VOTING	
Additional levy on meat products	Yes – Per kilogramm meat
Justification for the levy	Animal welfare in meat production
Usage of revenues from the levy	Investments in improvement of animal welfare in livestock farming
**Amount of the levy**	**Dependent** on husbandry level, no levy for plant-based alternatives

	**Levy by husbandry level**
**Level 1 (Stable housing)**	+2,15 EUR/kg
**Level 2 (Stable housing Plus)**	+1,76 EUR/kg
**Level 3 (Outside climate)**	+0,54 EUR/kg
**Level 4 (Premium)**	+0,31 EUR/kg
**Plant-based alternative**	+0,00 EUR/kg

If the majority of valid votes (>50%) is against this proposal, no [Script] will be implemented.

Do you vote for this proposal?

◯ Yes. I vote **for** the introduction of this levy.

◯ No. I vote **against** the introduction of this levy.

◯ I do not want to vote.

Next

0% 100%

awdiffr5

Please vote as if the shown proposal was **the only one available for vote**.

5. Proposal for an animal welfare levy:

PROPOSAL FOR VOTING	
Additional levy on meat products	Yes – Per kilogramm meat
Justification for the levy	Animal welfare in meat production
Usage of revenues from the levy	Investments in improvement of animal welfare in livestock farming
**Amount of the levy**	**Dependent** on husbandry level, no levy for plant-based alternatives

	**Levy by husbandry level**
**Level 1 (Stable housing)**	+3,23 EUR/kg
**Level 2 (Stable housing Plus)**	+2,64 EUR/kg
**Level 3 (Outside climate)**	+0,81 EUR/kg
**Level 4 (Premium)**	+0,47 EUR/kg
**Plant-based alternative**	+0,00 EUR/kg

If the majority of valid votes (>50%) is against this proposal, no [Script] will be implemented.

Do you vote for this proposal?

- Yes. I vote **for** the introduction of this levy.
- No. I vote **against** the introduction of this levy.
- I do not want to vote.

Next

0% 100%

awdiffr6

Please vote as if the shown proposal was **the only one available for vote**.

6. Proposal for an animal welfare levy:

PROPOSAL FOR VOTING	
Additional levy on meat products	Yes – Per kilogramm meat
Justification for the levy	Animal welfare in meat production
Usage of revenues from the levy	Investments in improvement of animal welfare in livestock farming
**Amount of the levy**	**Dependent** on husbandry level, no levy for plant-based alternatives

	**Levy by husbandry level**
**Level 1 (Stable housing)**	+4,30 EUR/kg
**Level 2 (Stable housing Plus)**	+3,52 EUR/kg
**Level 3 (Outside climate)**	+1,08 EUR/kg
**Level 4 (Premium)**	+0,62 EUR/kg
**Plant-based alternative**	+0,00 EUR/kg

If the majority of valid votes (>50%) is against this proposal, no [Script] will be implemented.

Do you vote for this proposal?

- Yes. I vote **for** the introduction of this levy.
- No. I vote **against** the introduction of this levy.
- I do not want to vote.

Next

0% 100%

clidiffr1

Please vote now:

Please vote as if the shown proposal was **the only one available for vote**.

1. Proposal for a climate levy:

PROPOSAL FOR VOTING	
Additional levy on meat products	Yes – Per kilogramm meat
Justification for the levy	Greenhouse gas emissions of meat production
Usage of revenues from the levy	Investments in climate protection
**Amount of the levy**	**Dependent** on meat type, no levy for plant-based alternatives

	**Levy by meat type**
**Beef**	+0,54 EUR/kg
**Lamb**	+0,44 EUR/kg
**Pork**	+0,14 EUR/kg
**Poultry**	+0,08 EUR/kg
**Plant-based alternative**	+0,00 EUR/kg

If the majority of valid votes (>50%) is against this proposal, no [Script] will be implemented.

Do you vote for this proposal?

◯ Yes. I vote **for** the introduction of this levy.

◯ No. I vote **against** the introduction of this levy.

◯ I do not want to vote.

Next

0% 100%

clidiffr2

Please vote as if the shown proposal was **the only one available for vote**.

2. Proposal for a climate levy:

PROPOSAL FOR VOTING	
Additional levy on meat products	Yes – Per kilogramm meat
Justification for the levy	Greenhouse gas emissions of meat production
Usage of revenues from the levy	Investments in climate protection
**Amount of the levy**	**Dependent** on meat type, no levy for plant-based alternatives

	Levy by meat type
**Beef**	+1,08 EUR/kg
**Lamb**	+0,88 EUR/kg
**Pork**	+0,27 EUR/kg
**Poultry**	+0,16 EUR/kg
**Plant-based alternative**	+0,00 EUR/kg

If the majority of valid votes (>50%) is against this proposal, no [Script] will be implemented.

Do you vote for this proposal?

- Yes. I vote **for** the introduction of this levy.
- No. I vote **against** the introduction of this levy.
- I do not want to vote.

Next

0% 100%

clidiffr3

Please vote as if the shown proposal was **the only one available for vote**.

3. Proposal for a climate levy:

PROPOSAL FOR VOTING	
Additional levy on meat products	Yes – Per kilogramm meat
Justification for the levy	Greenhouse gas emissions of meat production
Usage of revenues from the levy	Investments in climate protection
**Amount of the levy**	**Dependent** on meat type, no levy for plant-based alternatives

	Levy by meat type
**Beef**	+1,61 EUR/kg
**Lamb**	+1,32 EUR/kg
**Pork**	+0,41 EUR/kg
**Poultry**	+0,23 EUR/kg
**Plant-based alternative**	+0,00 EUR/kg

If the majority of valid votes (>50%) is against this proposal, no [Script] will be implemented.

Do you vote for this proposal?

- ◯ Yes. I vote **for** the introduction of this levy.
- ◯ No. I vote **against** the introduction of this levy.
- ◯ I do not want to vote.

Next

0% 100%

clidiffr4

Please vote as if the shown proposal was **the only one available for vote**.

4. Proposal for a climate levy:

PROPOSAL FOR VOTING	
Additional levy on meat products	Yes – Per kilogramm meat
Justification for the levy	Greenhouse gas emissions of meat production
Usage of revenues from the levy	Investments in climate protection
**Amount of the levy**	**Dependent** on meat type, no levy for plant-based alternatives

	**Levy by meat type**
**Beef**	+2,15 EUR/kg
**Lamb**	+1,76 EUR/kg
**Pork**	+0,54 EUR/kg
**Poultry**	+0,31 EUR/kg
**Plant-based alternative**	+0,00 EUR/kg

If the majority of valid votes (>50%) is against this proposal, no [Script] will be implemented.

Do you vote for this proposal?

- Yes. I vote **for** the introduction of this levy.
- No. I vote **against** the introduction of this levy.
- I do not want to vote.

Next

0% 100%

clidiffr5

Please vote as if the shown proposal was **the only one available for vote**.

5. Proposal for a climate levy:

PROPOSAL FOR VOTING	
Additional levy on meat products	Yes – Per kilogramm meat
Justification for the levy	Greenhouse gas emissions of meat production
Usage of revenues from the levy	Investments in climate protection
**Amount of the levy**	**Dependent** on meat type, no levy for plant-based alternatives

	Levy by meat type
**Beef**	+3,23 EUR/kg
**Lamb**	+2,64 EUR/kg
**Pork**	+0,81 EUR/kg
**Poultry**	+0,47 EUR/kg
**Plant-based alternative**	+0,00 EUR/kg

If the majority of valid votes (>50%) is against this proposal, no [Script] will be implemented.

Do you vote for this proposal?

- Yes. I vote **for** the introduction of this levy.
- No. I vote **against** the introduction of this levy.
- I do not want to vote.

Next

0% 100%

clidiffr6

Please vote as if the shown proposal was **the only one available for vote**.

6. Proposal for a climate levy:

PROPOSAL FOR VOTING	
Additional levy on meat products	Yes – Per kilogramm meat
Justification for the levy	Greenhouse gas emissions of meat production
Usage of revenues from the levy	Investments in climate protection
**Amount of the levy**	**Dependent** on meat type, no levy for plant-based alternatives

	Levy by meat type
**Beef**	+4,30 EUR/kg
**Lamb**	+3,52 EUR/kg
**Pork**	+1,08 EUR/kg
**Poultry**	+0,62 EUR/kg
**Plant-based alternative**	+0,00 EUR/kg

If the majority of valid votes (>50%) is against this proposal, no [Script] will be implemented.

Do you vote for this proposal?

- Yes. I vote **for** the introduction of this levy.
- No. I vote **against** the introduction of this levy.
- I do not want to vote.

Next

0% 100%

nonresp

You have chosen "I do not want to vote" **for at least one proposal**.
Why?

Please briefly state reasons for your abstention from voting.

Next

0% 100%

conseq

How much do you agree with the following statements?

	Not agree at all	Not agree	Rather not agree	Neither	Rather agree	Agree	Fully agree
Policymakers **should** consider the results of the vote in this survey when discussing the introduction of the proposed levy.	○	○	○	○	○	○	○
Policymakers **will** consider the results of the vote in this survey when discussing the introduction of the proposed levy.	○	○	○	○	○	○	○

Next

0% 100%

desirability

Please comment on the following human behaviors. What would you say: To what extent does the respective sentence apply to you personally?

	Does not apply at all	Does not apply	Rather does not apply	Neither	Rather applies	Applies	Fully applies
I've gotten too much change back before and said nothing.	◯	◯	◯	◯	◯	◯	◯
I am often unsure of my judgment.	◯	◯	◯	◯	◯	◯	◯
My first impression of people usually turns out to be correct.	◯	◯	◯	◯	◯	◯	◯
Please select the answer "Does not apply" here.	◯	◯	◯	◯	◯	◯	◯
I always know exactly why I like something.	◯	◯	◯	◯	◯	◯	◯
I am always honest to others.	◯	◯	◯	◯	◯	◯	◯
I have occasionally taken advantage of someone.	◯	◯	◯	◯	◯	◯	◯

Next

0% 100%

remark

Is there anything else you would like to tell us (e.g. praise, criticism, comments, etc.)?

Please keep in mind that we cannot answer you on this because answers in surveys are anonymous. Do not enter a phone number or other contact information here. These will be removed before analysis.

Next

Skip to:

0% 100%

Many measures on meat consumption are currently being discussed in German politics.

One proposal is to impose an **additional levy on meat products** (fresh meat, sausages and cold cuts).
Please read the following proposal carefully.

Proposal for an **animal welfare levy** on meat products:

- The government introduces a levy on meat products (fresh meat, sausages and spread).
- The levy is charged for every kilogram of meat sold - thus increasing the price you pay as a consumer.
- The **levy per kilogram** is based on the husbandry level.
- The following husbandry levels are considered: Level 1 (Stable housing), Level 2 (Stable housing Plus), Level 3 (Outside climate), Level 4 (Premium/Organic).
- The **amount** of the levy **is the same** for all husbandry levels. No levy is charged for plant-based alternatives.
- The figure below shows how the proposed levy, or price premium, compares **to each other** for each husbandry level. The exact amount of the levy will still be communicated to you in the course of the survey.

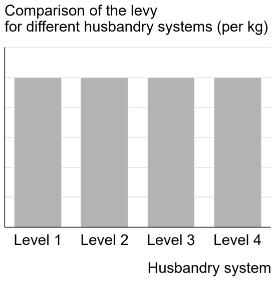

- If you would like to know what criteria must be met for each husbandry level, click on the image below:

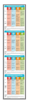

- The **revenue** from this levy will be invested in **improving animal welfare in livestock farming**.

Next

Skip to:

0% 100%

Many measures on meat consumption are currently being discussed in German politics.

One proposal is to impose an **additional levy on meat products** (fresh meat, sausages and cold cuts).
Please read the following proposal carefully.

Proposal for an **animal welfare levy** on meat products:

- The government introduces a levy on meat products (fresh meat, sausages and spread).
- The levy is charged for every kilogram of meat sold - thus increasing the price you pay as a consumer.
- The **levy per kilogram** is based on the husbandry level.
- The following husbandry levels are considered: Level 1 (Stable housing), Level 2 (Stable housing Plus), Level 3 (Outside climate), Level 4 (Premium/Organic).
- The **amount** of the levy **depends** on the husbandry level - **the higher the husbandry level, the lower the levy per kilogram**. No levy is charged for plant-based alternatives.
- The figure below shows how the proposed levy, or price premium, compares **to each other** for each husbandry level. The exact amount of the levy will still be communicated to you in the course of the survey.

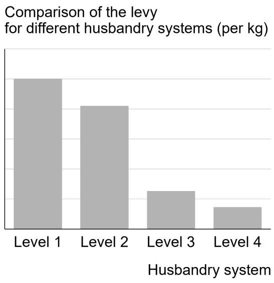

- If you would like to know what criteria must be met for each husbandry level, click on the image below:

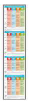

- The **revenue** from this levy will be invested in **improving animal welfare in livestock farming**.

Next

Skip to:

0% 100%

Many measures on meat consumption are currently being discussed in German politics.

One proposal is to impose an **additional levy on meat products** (fresh meat, sausages and cold cuts).
Please read the following proposal carefully.

Proposal for a **climate levy** on meat products:

- The government introduces a levy on meat products (fresh meat, sausages and spread).
- The levy is charged for every kilogram of meat sold - thus increasing the price you pay as a consumer.
- The **levy per kilogram** is based on the greenhouse gas emissions* of meat.
- The following meat types are considered: Beef, Lamb, Pork, Poultry.
- The **amount** of the levy **is the same** for all meat types. No levy is charged for plant-based alternatives.
- The figure below shows how the proposed levy, or price premium, compares **to each other** for each meat type. The exact amount of the levy will still be communicated to you in the course of the survey.

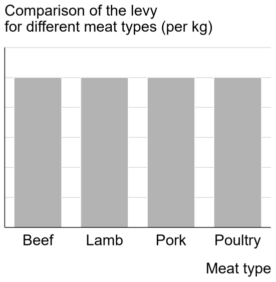

- If you would like to know how much and which greenhouse gas emissions are generated by each meat type, click on the image below:

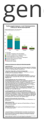

- The **revenue** from this levy will be invested in **climate protection**.

* In meat production the greenhouse gases carbon dioxide (CO_2), methane (CH_4) and nitrous oxide (N_2O) are produced.

Next

Skip to:

0% 100%

Many measures on meat consumption are currently being discussed in German politics.

One proposal is to impose an **additional levy on meat products** (fresh meat, sausages and cold cuts).
Please read the following proposal carefully.

Proposal for a **climate levy** on meat products:

- The government introduces a levy on meat products (fresh meat, sausages and spread).
- The levy is charged for every kilogram of meat sold - thus increasing the price you pay as a consumer.
- The **levy per kilogram** is based on the greenhouse gas emissions* of meat.
- The following meat types are considered: Beef, Lamb, Pork, Poultry.
- The **amount** of the levy **depends** on the meat type - **the lower the greenhouse gas emissions of a meat type, the lower the levy per kilogram**. No levy is charged for plant-based alternatives.
- The figure below shows how the proposed levy, or price premium, compares **to each other** for each meat type. The exact amount of the levy will still be communicated to you in the course of the survey.

Comparison of the levy
for different meat types (per kg)

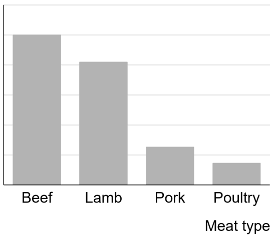

- If you would like to know how much and which greenhouse gas emissions are generated by each meat type, click on the image below:

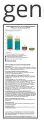

- The **revenue** from this levy will be invested in **climate protection**.

* In meat production the greenhouse gases carbon dioxide (CO_2), methane (CH_4) and nitrous oxide (N_2O) are produced.

Next

Skip to:

0% 100%

Now imagine that you can **participate in a Germany-wide referendum** on the animal welfare levy shown on the previous page.

On the next pages you will see **6 different proposals** one after the other. All proposals differ only in the amount of the levy.

As in a real referendum, you can **vote for or against** the proposal shown.

Please consider each proposal independently of the others. Vote as if the **proposal shown were the only one on the ballot**.

Your responses are important and should be heard.

After completion of the survey, we will send the results of this part of the survey to the **Committee on Food and Agriculture** and the **Committee on the Environment, Nature Conservation and Nuclear Safety** of the **German Bundestag**.

For each of the 6 proposals you are about to see, we inform the committees of the **proportion of respondents who voted for or against**. You can see the template for the letter to the committees below.

The letter is really sent to the two committees mentioned.

We only communicate the summarized results of the vote to the committees. **Your personal vote remains anonymous and secret.** No one, including the research team, can match your answers to your name or identity.

Template for the letter:

Skip to:

Logo Universität Hamburg

Deutscher Bundestag
Ausschuss für Ernährung und Landwirtschaft &
Ausschuss für Umwelt, Naturschutz und nukleare Sicherheit
Platz der Republik 1
11011 Berlin

3. Januar 2022

Betreff:
Studie zu Präferenzen der deutschen Bevölkerung für eine Tierwohlabgabe auf Fleischprodukte

Sehr geehrte Mitglieder des Ausschusses für Ernährung und Landwirtschaft und für Umwelt,
sehr geehrte Mitglieder des Ausschusses für Umwelt Naturschutz und nukleare Sicherheit,

wir sind ein Forschungsteam der Universität Hamburg und haben im Dezember 2021 eine wissenschaftliche Studie zu den Präferenzen der deutschen Bevölkerung für eine Abgabe auf Fleischprodukte durchgeführt.

Wir haben einer für die deutsche Bevölkerung repräsentativen Stichprobe an Teilnehmenden den folgenden Vorschlag für eine **Tierwohlabgabe auf Fleischprodukte** vorgestellt:

- Die Regierung führt eine Abgabe auf Fleischprodukte (frisches Fleisch, Wurst und Aufstrich) ein.
- Die Abgabe wird für jedes verkaufte Kilogramm Fleisch erhoben - sie erhöht damit den Preis, den Verbraucher:innen bezahlen.
- Die **Abgabe pro Kilogramm** orientiert sich an der Haltungsform der Tiere.
- Folgende Haltungsformen werden berücksichtigt: Stufe 1 (Stallhaltung), Stufe 2 (Stallhaltung Plus), Stufe 3 (Außenklima), Stufe 4 (Premium/Bio).
- Die **Höhe** der Abgabe ist von der Haltungsformstufe **abhängig** - **je höher die Haltungsformstufe, desto niedriger die Abgabe pro Kilogramm.** Für pflanzliche Alternativen wird keine Abgabe erhoben.
- Die **Einnahmen** aus dieser Abgabe werden **in die Verbesserung des Tierwohls in der Nutztierhaltung investiert**.

In der unten gezeigten Grafik sehen Sie den Prozentsatz der Teilnehmenden, die angegeben haben, dass Sie für die skizzierte Abgabe stimmen würden in Abhängigkeit von der Höhe in EUR/kg.

---- GRAFIK ----

[Die Grafik wird den Prozentsatz der Teilnehmenden, die für die sechs gezeigten Vorlagen mit „Ja. Ich stimme dafür." gestimmt haben, abbilden.]

Mit freundlichen Grüßen

Die Autoren der Studie
[Unterschriften]

Next

Skip to:

0% 100%

This survey asks people between the ages of 18 and 74 who live in Germany and maps the actual population structure of Germany for this age group.

What do you think?
Out of 100 respondents who were also asked the question about the development of **general meat consumption**, how many gave the answers "will decrease" and "will increase"?

BONUS

Please estimate 2 numbers on this page: one for each answer *"will decrease"* and *"will increase"*. The value for the answer *"will remain the same"* is calculated automatically in each line from your entries.
For each guess, if your estimated number differs from the correct number by 2 or less, you will receive an additional payment of **10 mingle points**. So you can get an additional payment of **max. 20 mingle points** on this page.

How many of 100 respondents gave the following answers?

	will decrease ↘	will increase ↗	will remain the same →	Sum
General meat consumption...			100	100

The value for *"will remain the same"* must be at least 0 and must not be negative. The sum must add up to 100.

Next

Skip to:

0% 100%

We also asked you how consumption by husbandry level would develop in Germany as a result of the animal welfare levy shown.

What do you think?
Out of 100 respondents who were also asked the questions about the development of **meat consumption by husbandry level**, how many gave the answers "will decrease" and "will increase"?

BONUS

Please estimate 4x2 numbers on this page: two for each husbandry level for the answers *"will decrease"* and *"will increase"* . The value for the answer *"will remain the same"* is calculated automatically in each line from your entries.
For each guess, if your estimated number differs from the correct number by 2 or less, you will receive an additional payment of **10 mingle points**. So you can get an additional payment of **max. 80 mingle points** on this page.

How many of 100 respondents gave the following answers?

The consumption of...

	will decrease ↘	will increase ↗	will remain the same →	Sum
Level 1 (Stable housing)	40	10	50	100
Level 2 (Stable housing Plus)	20	10	70	100
Level 3 (Outside climate)	10	45	45	100
Level 4 (Premium/Organic)	7	50	43	100

The value for *"will remain the same"* must be at least 0 and must not be negative. The sum must add up to 100.

Next

Skip to:

0% 100%

We also asked you how consumption by meat type in Germany would develop as a result of the climate levy shown.

What do you think?
Out of 100 respondents who were also asked the questions about the development of **meat consumption by meat type**, how many gave the answers "will decrease" and "will increase"?

BONUS

Please estimate 4x2 numbers on this page: two for each meat type for the answers *"will decrease"* and *"will increase"* . The value for the answer *"will remain the same"* is calculated automatically in each line from your entries.
For each guess, if your estimated number differs from the correct number by 2 or less, you will receive an additional payment of **10 mingle points**. So you can get an additional payment of **max. 80 mingle points** on this page.

How many of 100 respondents gave the following answers?

The consumption of...

	will decrease ↘	will increase ↗	will remain the same →	Sum
Beef			100	100
Lamb			100	100
Pork			100	100
Poultry			100	100

The value for *"will remain the same"* must be at least 0 and must not be negative. The sum must add up to 100.

Next
